# Coconut Oil as Lipid‐Based 3D‐Printable Sacrificial Material for the Facile Biofabrication of Precise Perfusable Microchannels and Freeform Hydrogel Structures

**DOI:** 10.1002/smsc.70371

**Published:** 2026-08-19

**Authors:** Salma Mansi, Susanne Halbherr, Franziska Möller, Anna Lotta Ernst, Sophie Bauer, Zinaida Kuznetsova, Myrto Karagiannakidou Samsarelou, Carolin A. Rickert, Dominik Heinrich, Zhaoyang Zhong, Diana M. Rojas‐González, Oliver Lieleg, Petra Mela

**Affiliations:** ^1^ TUM School of Engineering and Design Department of Mechanical Engineering Chair of Medical Materials and Implants Technical University of Munich Garching Germany; ^2^ Munich Institute of Biomedical Engineering (MIBE) Technical University of Munich Garching Germany; ^3^ Munich Institute of Integrated Materials Energy and Process Engineering Technical University of Munich Garching Germany; ^4^ TUM School of Engineering and Design Department of Materials Engineering Technical University of Munich Germany Garching Germany; ^5^ TUM Center for Functional Protein Assemblies (CPA) Technical University of Munich Garching Germany

**Keywords:** assisted printing, coconut oil, fugitive material, microchannel, sacrificial material, support bath, support material

## Abstract

Sacrificial materials provide temporary support in the biofabrication of 3D hydrogel constructs featuring cavities and overhangs or having slow polymerization preventing printability. Currently used sacrificial hydrogel inks take up water from the surrounding matrix hindering the shape fidelity of the final construct, for example microchannels with a flattened/irregular cross‐section. Furthermore, some inks require removal by chemical dissolution or mechanical forces with potential cytotoxic effects and damage of the main construct. Here, we propose lipid‐based sacrificial materials to address these limitations and introduce coconut oil as proof‐of‐concept hydrophobic, temperature‐responsive, cytocompatible, and printable sacrificial material that enables a cell‐friendly removal process. We demonstrate its suitability in the fabrication of microchannels with circular cross‐section and dimensional accuracy, which support functional endothelial layer formation, as demonstrated with dextran diffusion. Furthermore, we show the versatility of coconut oil by applying it as support material for the additive manufacturing of 3D macroscale hydrogel structures with overhangs and bridging elements as well as support bath for the fabrication of complex vascular structures in freeform embedded printing. The applicability with various hydrogels with different gelation mechanisms and the ease of use and the large availability at a low cost make the lipid‐based ink a promising material in biofabrication.

## Graphical abstract

Coconut oil is applied as sacrificial material for the fabrication of microchannels within hydrogel matrices and of macroscale 3D hydrogel constructs by supported and embedded printing. The hydrophobic ink does not take up water, thus guaranteeing shape fidelity. Furthermore, it is compatible with hydrogels with different crosslinking methods and offers easy removal at 37 °C.

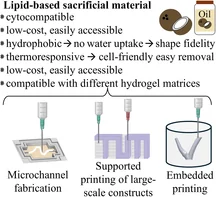

## 1 Introduction

Sacrificial materials play a crucial role in the fabrication strategies of different technological fields, from microelectromechanical systems (MEMS) to tissue engineering and regenerative medicine. These materials serve a temporary function in a multi‐step fabrication procedure after which they are removed, i.e., sacrificed, to obtain objects with overhangs or cavities. In general, requirements for these materials are the compatibility with previous and successive process steps and the possibility to selectively remove them without compromising the geometry they define. In biofabrication, sacrificial materials are used, for example, as leachable porogens in molding or electrospinning to create pores for cell infiltration [1, 2], as support structures in bioprinting [3], as support bath in embedded bioprinting such as freeform reversible embedding of suspended hydrogels (FRESH) [4, 5], and as dissolvable templates that will result in macroscale tubular constructs or [6] perfusable microchannels in polydimethylsiloxane (PDMS) devices [7] or hydrogel‐based constructs [8, 9, 10, 11]. Particularly, the fabrication of such microchannels has gained importance because of the increasing interest in the development of in vitro models [12, 13, 14] to study a variety of (patho)physiological processes, such as tumor intra/extravasation and selective crossing of the blood‐brain barrier, and to provide platforms for drug discovery [15, 16, 17, 18].

For these in vitro models, the fabrication of hydrogels containing cells sets stringent requirements on the sacrificial materials: cells should not be exposed to elevated temperatures or toxic organic solvents, and the sacrificial material should be removed rapidly to prevent prolonged nonphysiological conditions, including uncontrolled diffusion of the sacrificial material or solvent into the gel.

Thermoresponsive hydrogels, such as Pluronic F‐127 and gelatin, are typically used as they can be directly printed on cell‐laden layers, thereby taking advantage of the freedom of design characteristic of additive manufacturing [19, 20, 21]. Furthermore, once embedded in a matrix, they can be removed with a cell‐compatible temperature change to form the microchannel, thus eliminating the drawbacks associated with other hydrogels, such as agarose and alginate. For those inks, the removal process is still mechanical, e.g., by aspiration or manual pulling [22, 23, 24, 25], with the associated risk of damaging the surrounding hydrogel matrix besides the limitation to simple geometries such as straight and open‐end channels. Alternatively, 3D‐printed alginate channel templates were dissolved by the addition of calcium chelators, which, however, depending on their dosage and on the duration of the removal process, can negatively affect cell viability in the surrounding construct [26, 27]. The use of hydrogels as sacrificial materials for microchannel fabrication is intrinsically problematic as they take up water from the surrounding matrix, due to their hydrophilic nature, and, by doing so, lose their shape, with the consequence that the geometry they define is not precise anymore [28, 29]. Poor shape fidelity means that the cross‐section of the channels is not circular but has an irregular, flattened shape instead, as seen with gelatin that was used for creating vascularized structures in a collagen matrix [21, 30] or with Pluronic F‐127 for creating channels embedded in gelatin–fibrin or extracellular matrix (ECM) hydrogels [11, 15, 17, 20]. Additional drying steps prior to printing would be needed to limit such issue, at the cost of introducing a manual step with low reproducibility [15, 20].

However, it has been shown that the cross‐sectional geometry of microchannels plays an important role in cellular behavior. For instance, Esch et al. showed differences in endothelial cell focal adhesions on circular and square channels made of PDMS [31] and Jouybar et al. [32] reported that flow conditions affected the endothelial cells differently in circular and rectangular channels. The fabrication of PDMS microchannels can rely on sacrificial materials with high melting temperatures and requiring harsh or long removal processes [33, 34].

Besides guaranteeing shape fidelity, an ideal sacrificial material should possess suitable viscoelastic properties for printability, be processable at temperatures compatible with the presence of cells, and be easily removable, possibly in a short time. Furthermore, it should not be cytotoxic or leave residues that hinder cell attachment and should be compatible with a variety of hydrogels, i.e., it should not interfere with their crosslinking mechanisms. Accessibility and low cost, as well as easy and time‐efficient preparation, are additional properties that would make the material attractive and valuable for a large number of research groups.

Here, we propose the use of lipid‐based sacrificial materials to avoid water uptake because of their hydrophobic nature and obtain high shape fidelity. Specifically, we introduce coconut oil as model system that meets all the above requirements. Coconut oil is an edible oil extracted from the kernel of the coconut (Cocos nucifera) and is composed predominantly of saturated triglycerides rich in medium‐chain fatty acids, primarily lauric acid. It exhibits a high oxidative stability and a thermally reversible solid–liquid phase transition, remaining solid below approximately 24–26 °C. Common applications encompass nutritional, pharmaceutical, and cosmetic products [35, 36, 37, 38]. Here, we investigate its rheological properties, printability and removal, and cytocompatibility, as well as its applicability in three different fabrication scenarios, specifically to obtain perfusable endothelialized microchannels, large‐scale structures with bridging and overhangs, and complex 3D structures in embedded printing. Additionally, we verify its compatibility with hydrogel matrices with different crosslinking mechanisms.

## 2 Results and Discussion

**FIGURE 1:**
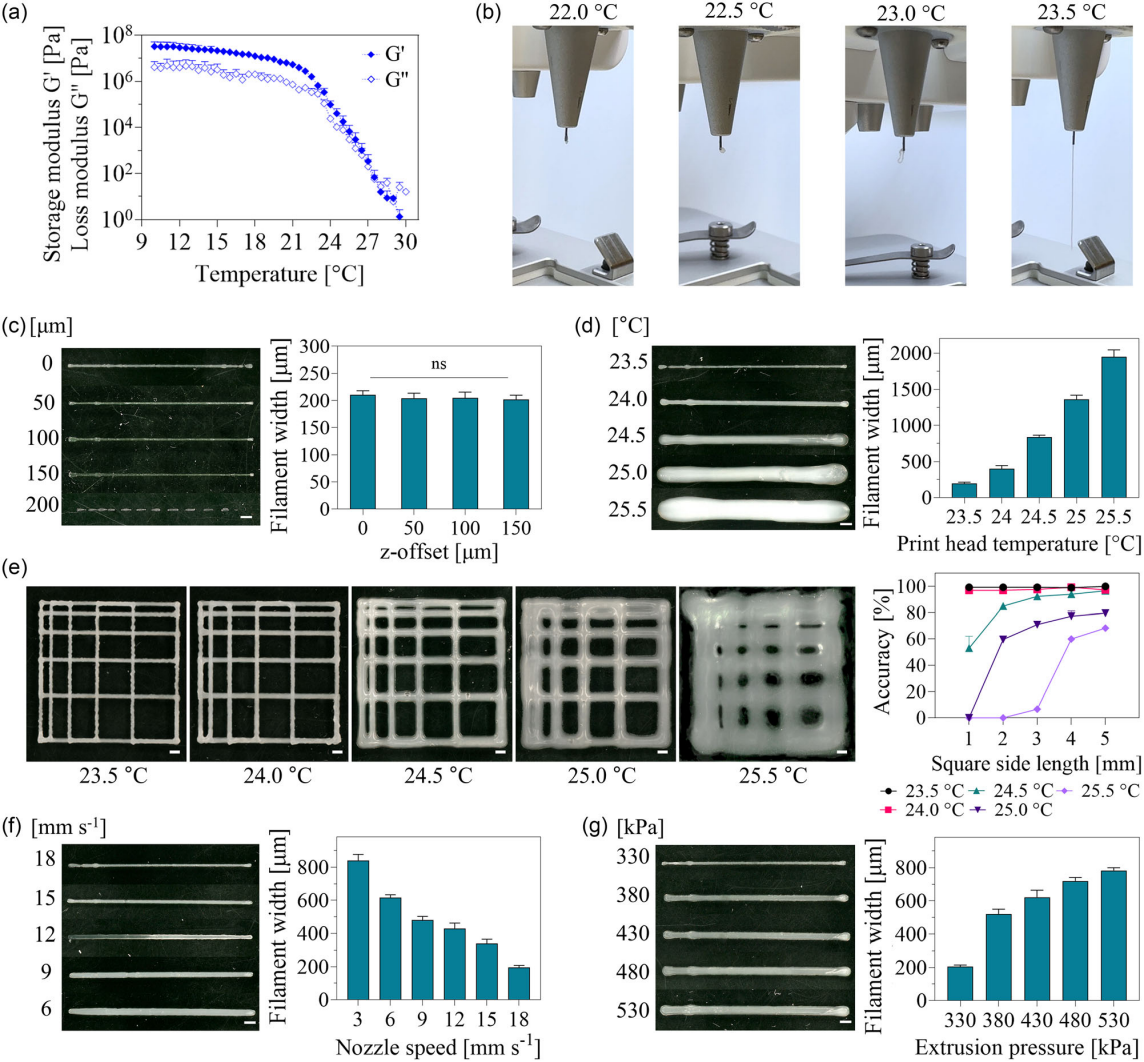
Printability evaluation. (a) Temperature sweep at 1 Hz frequency. (b) Ink extrudability test at print head temperatures between 22 and 23.5 °C and an extrusion pressure of 330 kPa. Visualization and quantification of the filament width as a function of (c) the *z*‐offset and of (d) the print head temperature. (e) Pore accuracy test at different print head temperatures determined on the grid patterns with theoretical pore sizes of 1, 4, 9, 16, and 25 mm^2^. Visualization and quantification of filament width as a function of (f) the nozzle speed and of (g) the extrusion pressure. If not stated because kept constant, the printing parameters were as follows: extrusion pressure of 330 kPa, *z*‐offset of 150 μm, print head temperature of 23.5 °C, and nozzle speed of 18 mm s^−1^. All experiments were performed with a 27G needle. Scale bar (c–g): 1 mm. Values represent mean ± standard deviation obtained from (a) *n* = 3, (c) *n* = 10, (d,f,g) *n* = 20, and (e) *n* = 3 independent samples. If not visible, the error bars are smaller than the symbol size.

A rheological evaluation of the ink was conducted to determine its temperature‐dependent behavior and define the printing conditions by performing a temperature sweep between 10 and 30 °C. Up to 22 °C, the behavior of the material was clearly elastically dominated, with a slow decline in material stiffness, while a further increase of temperature resulted in a reduction in stiffness by five orders of magnitude, with the difference between G′ and G″ steadily decreasing (Figure 1a). Beyond 28 °C, the material liquefied, and its response became clearly viscously dominated. Within the temperature range of 22–25 °C, the material exhibited a shear thinning behavior (Figure S1a) and a decreasing yield stress with increasing temperature (Figure S1b,c). To define the printing temperature, the ink extrudability was assessed starting at a temperature of 22 °C by evaluation of filament formation, which, using a 27G needle, was not achieved for temperatures below 23.5 °C with an extrusion pressure of 330 kPa, (Figure 1b), nor when increasing the pressures to 550 or 700 kPa (Figure S2). Hence, for the following printability study, print head temperatures from 23.5 to 25.5 °C were examined. A print bed temperature of 10 °C was chosen to ensure fast solidification of the printed constructs upon cooling and thus print fidelity.

Figure 1c shows the deposited filament as a function of *z*‐offset, defined as the vertical distance between the needle tip and the print bed after subtracting the needle's inner diameter, as implemented by the printer. Increasing the *z*‐offset from 0 to 150 µm resulted in continuous filaments with a small but nonsignificant decrease in width, while a *z*‐offset of 200 µm produced a discontinuous filament. As we intended to print on hydrogel matrices, which might not be perfectly flat, we chose 150 µm to avoid a possible interference between the needle and the deposited filament without the risk of printing too far away from the surface.

Next, we printed single filaments and grids at print head temperatures between 23.5 and 25.5 °C and a print bed temperature of 10 °C (Figure 1d,e). This rather narrow temperature range had a significant effect on the filament width, with a tenfold increase (Figure 1d), and, as a consequence, on the accuracy of the grids (Figure 1e). The highest accuracy values of the pore areas were achieved at a temperature of 23.5 °C, corresponding to sharp‐cornered squares (Figure 1e); therefore, 23.5 °C was chosen as the optimal print head temperature. Because of the narrow temperature window for printability, the bioprinter's ability to precisely maintain the print head temperature throughout the printing process is essential.

To examine the influence of other printing parameters on the filament width, nozzle speed and extrusion pressure were varied. The filament width decreased with increasing nozzle speed and decreasing extrusion pressure (Figure 1f,g). The smallest filaments with a mean diameter of 195 ± 12 µm were achieved with a nozzle speed of 18 mm s^−1^, an extrusion pressure of 330 kPa, and a temperature of 23.5 °C (Figure 1g). Thus, these parameters were chosen as printing parameters for all following experiments.

**FIGURE 2:**
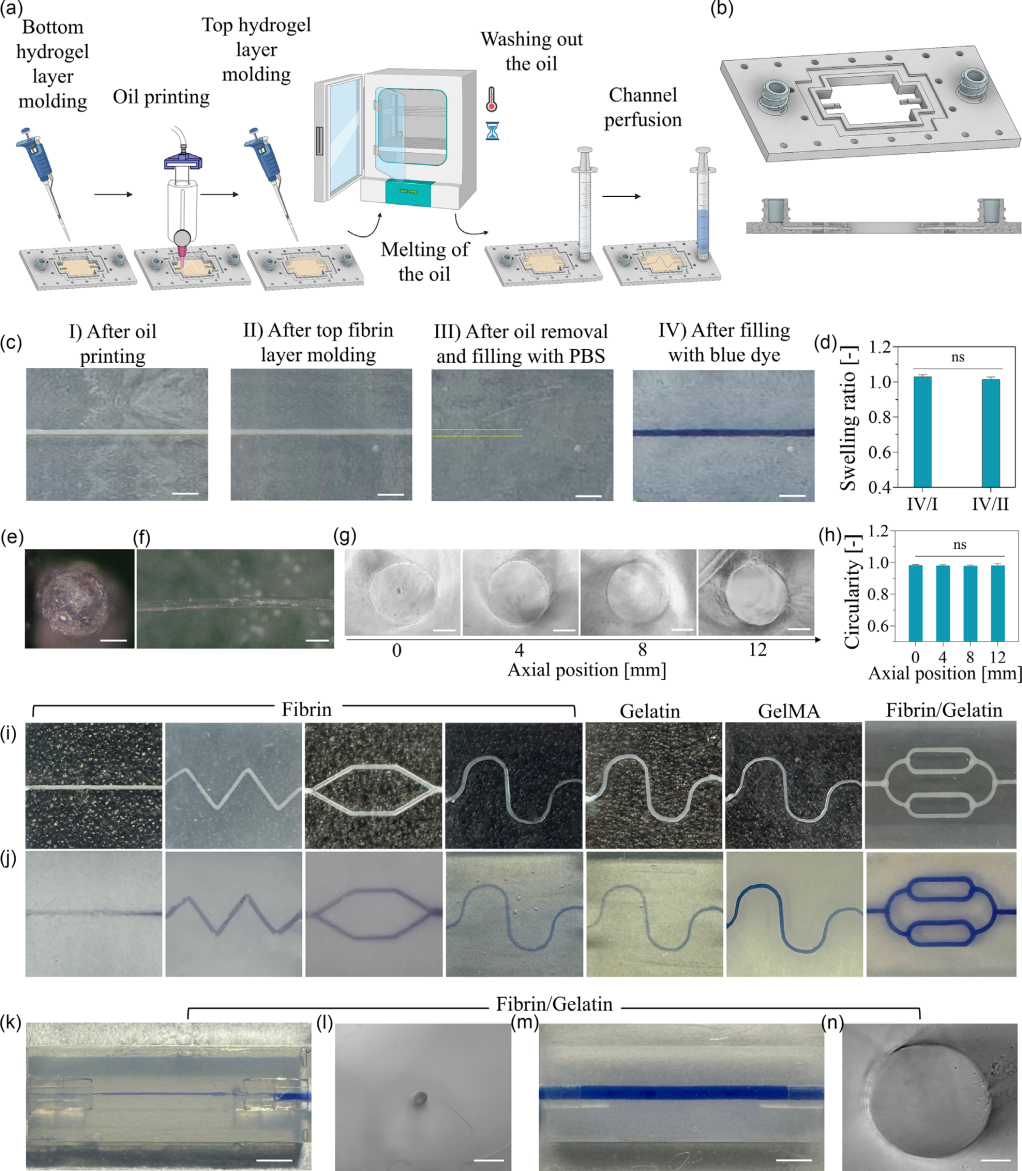
Microchannel fabrication. (a) Schematic of the fabrication process. (b) Design of the flow chamber for the perfusion of the channels. (c) Images of (I) the filament directly after printing on a fibrin layer using a 27G needle, (II) the filament after embedding by molding the top fibrin layer, (III) the channel after ink removal by washing and filling with PBS, and (IV) the channel after filling with blue dye. (d) Swelling ratio of the resulting channel filled with blue dye to the printed filament (IV/I) and to the embedded filament (IV/II). Values represent mean ± standard deviation obtained from *n* = 4 independent samples. (e) Cross‐sectional and, (f) longitudinal view of the negative replica of the channel in PEGDA. (g,h) Microchannel cross‐sectional circularity. (g) Phase contrast images of the cross‐section of a microchannel produced in a fibrin/gelatin matrix with a 20G needle, and (h) the calculated circularity of the cross‐sections along the channels’ length (12 mm). Values represent mean ± standard deviation as obtained from *n* = 4 independent samples. (i) Pictures of the coconut oil filaments printed with different patterns and embedded in matrices of fibrin, gelatin, and gelatin methacryloyl, and (j) of the corresponding perfused microchannels resulting from the removal of the coconut oil, visualized with blue dye. (k–n) Microchannel diameter range. Longitudinal and cross‐sectional views of channels produced in a fibrin/gelatin matrix with a (k–l) 34G and (m–n) 18G needle. Scale bars: (c) 800 μm, (e) 100 μm, (f) 500 μm, (g) 200 μm, (k,m) 2 mm, (l,n) 200 μm.

The fabrication scheme to produce microchannels embedded in a hydrogel matrix relies on the use of the coconut oil as sacrificial material as depicted in Figure 2a: a first hydrogel layer is molded inside a flow chamber, the coconut oil filament is printed onto it, and a second hydrogel layer is molded to embed the filament, which is then liquified and washed out at 37 °C, leaving a perfusable channel. We designed a flow chamber (Figure 2b) to enable the perfusion of the channels through the connection to a flow system. The inlet and outlet of the chamber were designed with external Luer‐lock fittings and internal semicylindrical guiding structures, on which the end portions of the coconut filament are printed to ensure continuity between the microchannel and the tubing of the flow system.

We next evaluated whether the printed filaments maintained their geometry when embedded in hydrogels. We chose fibrin as a representative hydrogel matrix with high water content, which is used in a wide range of applications in cell‐based tissue engineering, including vascular constructs [39, 40, 41, 42, 43, 44]. The swelling behavior of the printed coconut filaments in contact with hydrogels was determined by evaluating the width of the sacrificial filaments immediately after printing (I), after embedding the filaments by molding the top hydrogel layer (II), and after filament removal and channel filling with a blue dye (IV) for visualization (Figure 2c(I–IV)). A swelling ratio around 1 (Figure 2d) indicated no water uptake by the filament, as expected from a lipid‐based material. To examine the channel cross‐section, we created a negative replica of the channel by filling it with polyethylene glycol diacrylate (PEGDA) containing lithium phenyl (2,4,6‐trimethylbenzoyl) phosphinate (LAP) as photoinitiator and exposing it to UV. The crosslinked filament was then pulled out of the channel and examined by light microscopy. Figure 2e shows the round cross‐section of the negative replica, in contrast to the flattened/irregular channel geometries obtained with commonly used sacrificial inks such as Pluronic F‐127, gelatin, or Carbopol, because of their spreading and water uptake from the surrounding hydrogel matrix [16, 17, 21, 30, 45]. The side view of the negative replica (Figure 2f) showed a width of 267 ± 17 µm (*n* = 4 channels, *i* = 2) over the length of the channel. The increase of the channels’ width can be explained by the pressure applied to the soft fibrin gel during the injection of the viscous PEGDA for the negative replica fabrication, performed manually using a syringe connected to the chamber Luer inlet. Although care was taken in this step, due to the higher viscosity of the PEGDA solution compared to phosphate buffered saline (PBS), higher forces were needed to fill the lumen leading to the observed channel widening. Alternatively, we characterized the cross‐sectional circularity of microchannels produced in a fibrin/gelatin matrix using a 20G needle (inner diameter: 610 µm) by carefully slicing the hydrogel with a sharp scalpel and imaging the hydrogel sections. We determined a diameter of 606 ± 9 µm and circularity of 0.98 ± 0.01 (*n* = 4) showing dimensional and geometrical accuracy along the channel's axial length (Figure 2g,h), in strong contrast with what is reported in studies performed with hydrogel‐based materials such as gelatin, Pluronic F‐127, agarose, and xanthan gum [15, 16, 17, 20, 21, 46, 47].

To further explore the applicability of coconut oil, microchannel fabrication with different geometries, e.g., zigzag, bifurcation, and serpentine, was demonstrated in fibrin (Figure 2i,j), and, to evaluate the compatibility with other crosslinking mechanisms, the serpentine geometry was produced in gelatin enzymatically crosslinked with transglutaminase and in gelatin methacryloyl (GelMA) crosslinked by UV irradiation (Figure 2i,j). A more complex vascular network was fabricated in a fibrin/gelatin matrix as shown in Figure 2i,j.

Based on the needle sizes compatible with the printer (34–18 G, inner diameter: 85–840 µm), the minimum and maximum channel diameters were examined. Figure 2k–n shows the longitudinal and cross‐sectional view of microchannels produced in a fibrin/gelatin matrix with the 34G (Figure 2k,m) and 18 G (Figure 2l,n) needles. When using the 18G needle, a pressure of 55 kPa and a print head temperature of 23.5 °C were applied, and a diameter of 837 ± 9 µm (*n* = 4) was achieved. For the 34 G needle, an extrusion pressure of 700 kPa and a print head temperature of 24.5 °C to slightly decrease the viscosity were used, leading to a diameter of 86 ± 4 µm (*n* = 4). The produced microchannel diameters in our study are within the range achieved with extrusion‐based sacrificial printing in literature spanning 45–1000 µm [28, 48, 49, 50, 51].

**FIGURE 3:**
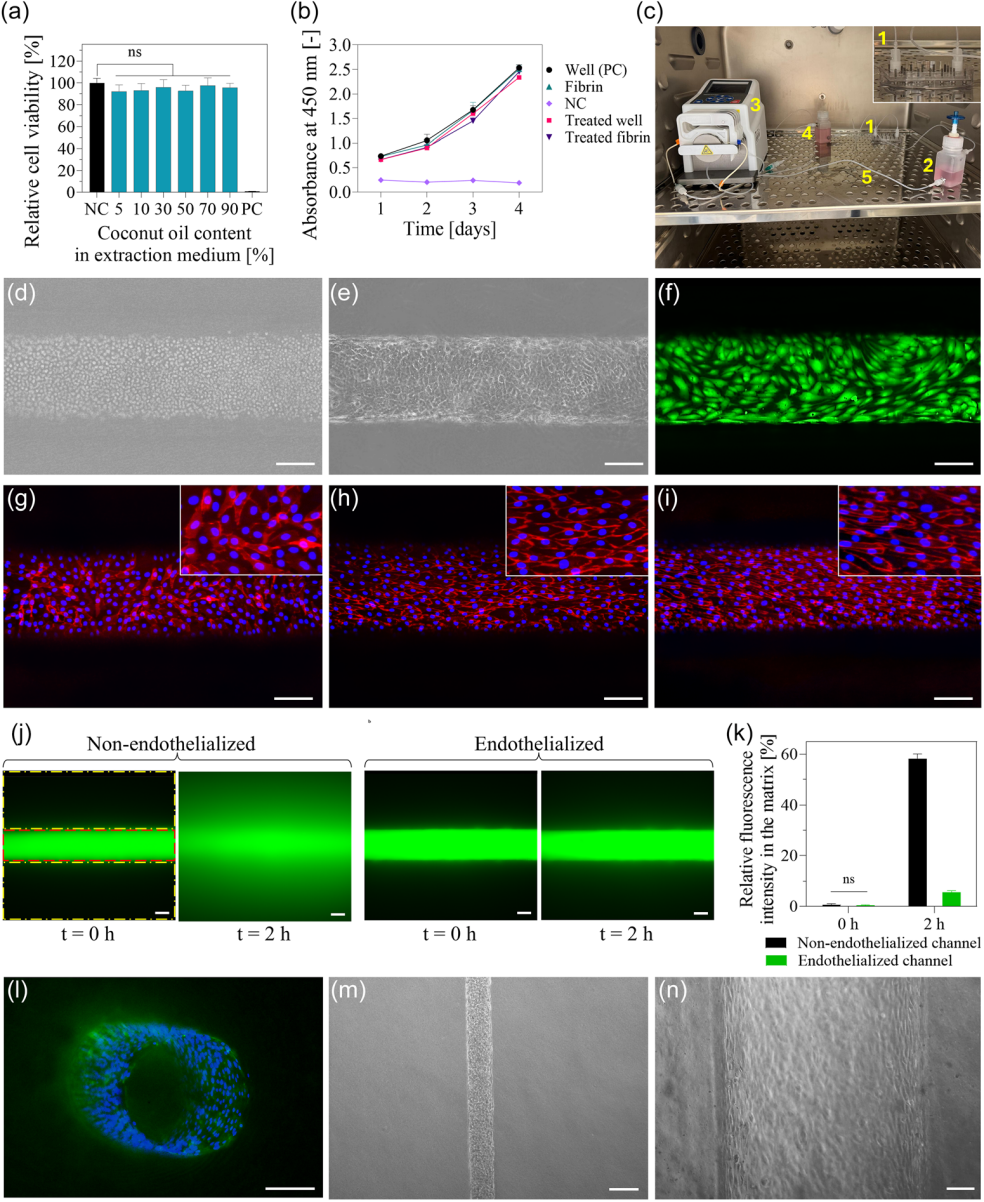
Endothelialization of microchannels. (a) Relative cell viability of Hs27 fibroblasts after incubation in the eluates created from the oil/medium mixtures at different oil concentrations for 72 h. Untreated medium served as the negative control (NC) and medium with 1% triton X‐100 served as the positive control (PC). (b) XTT proliferation assay of Hs27 fibroblasts over 4 days seeded on treated (i.e., oil‐covered and PBS‐rinsed) wells and fibrin gels. Untreated wells served as positive control (PC) and untreated wells in medium with 1% triton X‐100 served as the negative control (NC). Values represent mean ± standard deviation obtained from *n* = 5 independent samples. (c) Illustration of the flow system including the (1) flow chamber, (2) medium reservoir, (3) peristaltic ump, (4) compliance chamber, and (5) tubing. Inset: magnified view of (1) the flow chamber. (d) Phase‐contrast image of a channel in fibrin gel produced with a 27 G needle immediately after seeding the endothelial cells, and (e,f) phase‐contrast and live/dead staining images of the endothelialized channel after overnight perfusion at 0.1 dyne cm^−2^. Images of CD31 immunostaining (red) and DAPI staining (blue) of the endothelialized channels (g) on day 2 after overnight perfusion for 16 h at 0.1 dyne cm^−2^, (h) then on day 3 after overnight perfusion for 16 h at 2 dyne cm^−2^, and (i) after further 72 h at 10 dyne cm^−2^. (j) Images of the nonendothelialized and endothelialized channels (as in (k)) incubated with 70 kDa FITC dextran at 0 and 2 h. The fluorescence intensity was determined in the matrix surrounding the channel (yellow‐framed regions) and inside the channel (red‐framed region) to determine (k) the relative fluorescence intensity of the endothelialized and nonendothelialized channels. Values represent mean ± standard deviation obtained from *n* = 4 independent experiments. (l) Cross‐sectional view of an endothelialized microchannel (as in (k)) stained for actin (green) and DAPI (blue). (m,n) Microchannel diameter range. Phase contrast images of endothelialized microchannels produced in a fibrin/gelatin matrix with (m) a 34G and (n) an 18G needle. Scale bar (d–n): 100 μm.

We proceeded with the evaluation of the cytocompatibility of the coconut oil to ensure its suitability for biomedical applications. The relative viability of Hs27 fibroblasts incubated in the eluates from different medium/oil mixtures with 5%–90% v/v coconut oil was determined according to ISO 10993 (Figure 3a), resulting in values higher than 70% for all eluates, thereby demonstrating the oil's cytocompatibility. The application of coconut oil as sacrificial material also requires the unhindered cell proliferation on surfaces previously in contact with the ink. Therefore, the proliferation of Hs27 fibroblasts was investigated on surfaces that were covered with coconut oil and subsequently rinsed with PBS, to simulate the microchannel fabrication process. The proliferation of cells seeded on wells and fibrin gels that had been in contact with coconut oils was not statistically different from that on untreated wells and gels (Figure 3b), showing increasing surface coverage over a cultivation period of 4 days on all sample surfaces (Figure S3).

The endothelialization and perfusion of the microchannels was conducted with the flow system depicted in Figure 3c. Endothelial cells were introduced in the microchannel (Figure 3d) incubated 6 h under static condition and subsequently overnight under low perfusion (0.1 dyne cm^−2^) to reach the formation of a confluent layer (Figure 3e) as verified with calcein‐AM (Figure 3f) and CD31 staining (Figure 3g). When subjected to increased flows resulting in shear stresses of 2 and 10 dyne cm^−2^, the cells assumed an elongated morphology and aligned in the direction of the flow, in agreement with previous studies that reported cell alignment at shear stresses between 5 and 17 dyne cm^−2^ in fabricated microchannels [47, 52, 53, 54]. We also observed clear intercellular junctions (Figure 3h–i), as well as higher endothelial cell density and increased nuclear orientation parallel to the flow direction (Figure S4) [47, 52, 53, 54].

The formation of intercellular junctions plays an important role in the permeability of the endothelium, regulating the passage of water, solutes, and larger molecules between the circulating fluid and the surrounding tissues [55]. The barrier function of the endothelialized channels was investigated by incubating nonendothelialized and endothelialized channels (prepared as in Figure 3h) with fluorescein isothiocyanate (FITC)‐labeled dextran with a molecular weight of 70 and 250 kDa (Figures 3j and S5a) and by evaluating the diffusion of the dextran into the surrounding hydrogel matrix over a 2 h period. A 70 kDa FITC‐labeled dextran was chosen as it has a similar molecular weight to the abundant human blood plasma protein albumin (approx. 66–67 kDa), typically not crossing the endothelium, which has a cut‐off size around 60–70 kDa [56]. A 250 kDa FITC‐labeled dextran represents larger molecules such as drugs that can cross a leaky endothelium, e.g., as seen with tumor blood vessels [55]. The fluorescence intensity in the matrix relative to that inside the channel (Figures 3k and S5b) revealed a significantly higher increase for the nonendothelialized channel after 2 h compared to the endothelialized one, indicating a functional endothelial barrier. Our results are in agreement with previous studies reporting lower levels of dextran diffusion from endothelialized vessels compared to nonendothelialized ones [16, 17, 47, 57, 58]. The presence of a functional endothelial barrier is a condition needed to study tumor‐induced or inflammatory vascular disruption in the frame of disease modeling and drug development studies, which we aim to address in the near future.

The cross‐sectional view of an endothelialized microchannel, as used for the dextran diffusion experiment, stained for actin and DAPI confirmed 3D coverage of the channel surface area (Figure 3l). We further showed that the endothelialization process is applicable to channels produced with the smallest (34 G) and largest (18 G) needle sizes compatible with the printer (Figures 3m,n and S6).

**FIGURE 4:**
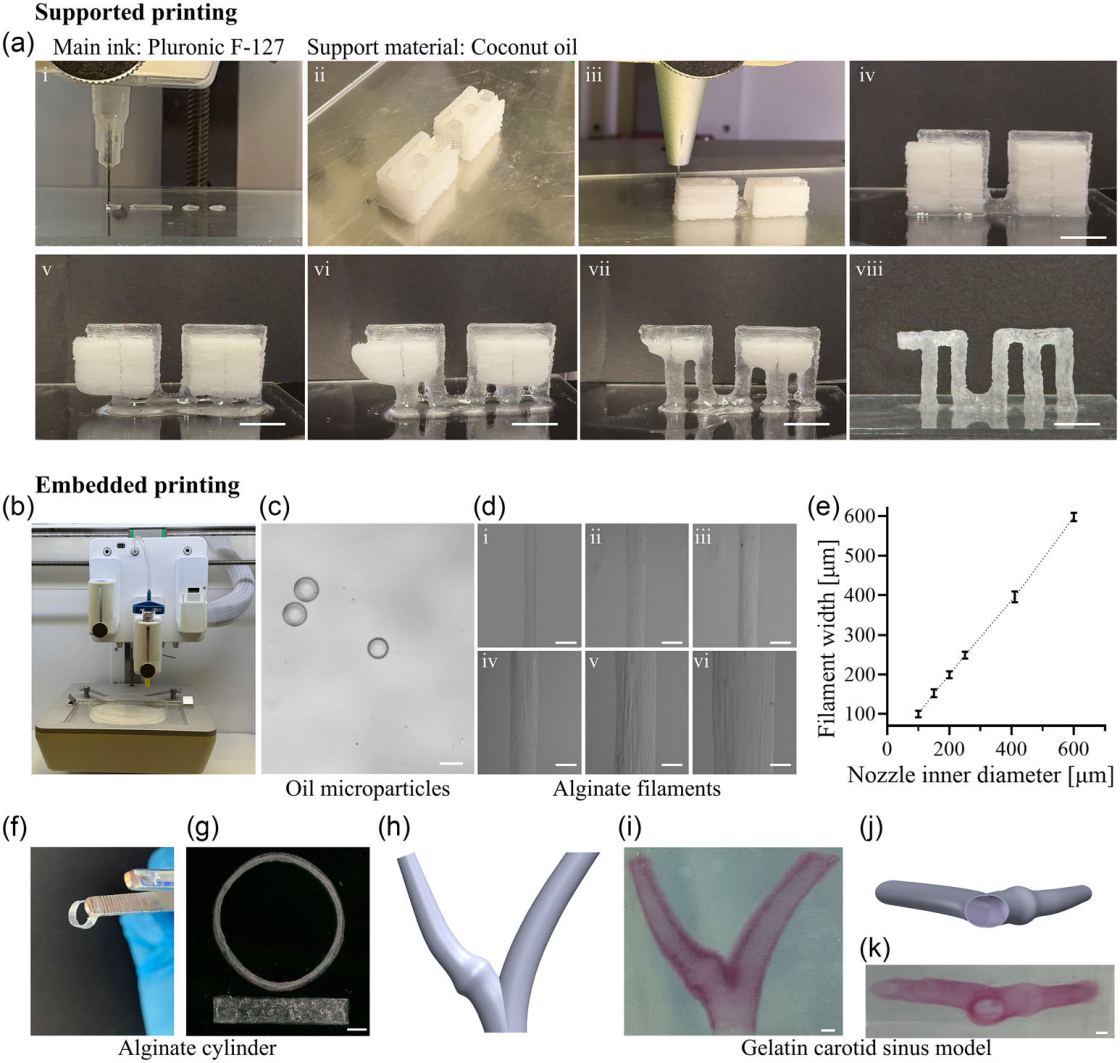
Applications of coconut oil as sacrificial material. Supported printing: (a) Printing of the Pluronic F‐127‐based TUM university logo using support structures from coconut oil. The images show the logo during printing (i–iii), still embedded in the coconut ink (iv), partially released from the liquifying ink (v–vii photos taken from the captured video) and as self‐standing object with overhang and bridging of vertical parts with aspect ratio of 5.4 (viii). Embedded printing: (b) image of the prepared coconut oil support bath for embedded printing in a Petri dish placed on the print bed. (c) Image of the bath microparticles. (d) Images of alginate filaments printed with different nozzles, specifically with (i) 32 G, (ii) 30 G, (iii) 27 G, (iv) 25 G, (v) 22 G, and (vi) 20 G. (e) Filament width in relation to the nozzle inner diameter. Values represent mean ± standard deviation obtained from *n* = 5 independent samples. (f,g) Images of the alginate cylindrical construct (3 × 10 mm). (h,j) 3D model and (i,k) printed construct of a carotid sinus (free CAD model available on grabcad.com). Scale bar: (a) 10 mm, (c) 20 µm, (d) 200 µm, (g,i,k) 2 mm.

We proceeded to assess the performance of the coconut oil as a sacrificial material in another fabrication scenario, specifically as support material to produce large structures with overhangs and bridging sections. We chose the TUM logo with a letters’ height of 20.6 mm and cross‐section of 3.8 × 3.8 mm, resulting in an aspect ratio of 5.4, to be realized with Pluronic F‐127 as a model for hydrogels. The Pluronic F‐127 and the coconut oil were printed using two extrusion print heads (Figure 4a(i–iii)), and the oil was subsequently liquified at 27 °C and removed. Figure 4a(iv) shows the TUM logo still supported by the solidified coconut oil, and Figure 4a(v–viii) the removal of the support till the final result was achieved, with overhanging and bridging structures in the letters “T” and “M,” which would not be possible without support. The logo (*n* = 3) was printed with an accuracy of 94.9 ± 3.2% and 99 ± 0.7% for the total height and length of the logo, respectively, and 93.3 ± 5.4% for the letter widths. The working temperatures of coconut oil are compatible with the gelation conditions of many hydrogels, such as gelatin, alginate, fibrin, and gelatin methacryloyl hydrogels. The direct delivery of the crosslinking agent can be realized, for example, by localized spraying or nebulization, or via a third print head [59].

Printing of larger scale and high aspect ratio structures as in Figure 4a shows that the temperature control of the print head and the print bed guarantees the correct printing conditions in the build volume as further demonstrated in Figure S7. Hypothermic conditions are compatible with cell viability and are commonly used, for example, for transport. It is common in bioprinting to print with cell‐laden bioinks at ambient temperature or lower, for example on print beds cooled to temperatures between 7 and 15 °C [60, 61]. As can be appreciated from the rheological characterization, the print bed temperature could, if necessary, be increased. We show that up to 15 °C no significant change in filament diameter was observed (Figure S8).

As third fabrication scenario, we looked at the fabrication of complex, freeform hydrogel structures in embedded printing. This approach is particularly interesting for low‐viscosity and slow polymerizing hydrogels as the support bath constrains the precursor in a specific geometry and, at the same time, can support crosslinking by diffusion. Constructs ranging in size from a few hundred microns to several tens of centimeters can be obtained [4, 5, 62]. We specifically investigated the suitability of the coconut oil for the FRESH method, for which gelatin microparticles are typically used [4, 5]. To this end, we created a coconut oil‐based emulsion and stabilized it with xanthan gum (Figure 4b), which resulted in small microparticles with a size of ca. 21.6 ± 3 µm (Figure 4c), comparable in size to the gelatin microparticles reported in the literature with sizes ranging between 25 and 65 μm [4, 5]. We proceeded with the proof‐of‐concept validation by printing alginate filaments and hollow cylinders (Figure 4d–g) in the support bath containing calcium chloride as crosslinker. Filaments printed using nozzles with inner diameters between 100 and 600 µm exhibited widths that accurately reproduced the nozzle gauges (Figure 4d,e), while a nozzle with a diameter of 100 µm was used to print hollow cylinders with diameter of 10 mm and height of 3 mm (Figure 4f,g). Furthermore, a carotid sinus model (Figure 4h,j) was printed with an 8 wt% gelatin solution and enzymatically crosslinked with transglutaminase (Figure 4i,k), emphasizing the bath's applicability for models with more complex geometries, such as bifurcations and vessel dilations, as well as its suitability for different materials. All structures were removed from the bath upon liquifying the coconut oil at 37 °C. This temperature makes the bath advantageous when printing cell‐laden constructs as it avoids temperature‐induced cell damage. Another advantage of the bath is the ease of preparation, which could be completed within 1–2 h, whereas other approaches using, e.g., gelatin‐ or alginate‐based support baths require at least 12–14 h [4, 5, 62, 63, 64]. However, a clear disadvantage with respect to these materials is the lack of transparency of the bath hindering the direct monitoring of the printing process.

While we validated the application of coconut oil with one commercial product, other similar low‐cost products are also processable, provided that the printing parameters are specifically defined, as preliminarily shown in the supplementary information (Table S1, Figure S9).

## 3 Conclusion

In this study, coconut oil was introduced as a lipid‐based sacrificial material and its versatility was demonstrated for different fabrication approaches and materials, specifically for the fabrication of microchannels embedded in hydrogel matrices, for supported printing of larger 3D constructs, and for freeform embedded printing. The ink satisfies the requirements for an ideal 3D‐printable sacrificial material by possessing suitable viscoelastic properties and being cytocompatible. Its easy and quick removal at 37 °C avoids exposing the cells in the hydrogel matrix to nonphysiological conditions and does not hinder cell attachment and growth. Furthermore, the ink is compatible with a variety of hydrogel matrices, with different crosslinking mechanisms, as shown for fibrin, gelatin, GelMA, and alginate. This study addresses current limitations of sacrificial inks related to shape fidelity and offers an alternative material with advantageous properties, including uncomplicated and fast preparation, as well as easy accessibility and low cost. Although demonstrated here with coconut oil, the concept of using lipid‐based sacrificial materials can potentially be extended to other readily available (food) products, such as cocoa butter, ghee, leaf lard, and palm oil.

## 4 Materials and Methods

### 4.1 Rheology

Prior to each experiment, the samples were equilibrated in a shaking incubator at 23.5 °C for 24 h. The rheological measurements were conducted on a commercial shear‐rheometer (MCR302, Anton Paar, Graz, Austria) in a plate–plate configuration (P‐PTD200 and PP25, Anton Paar) with a gap of 300 µm and a sample volume of 300 µl. The system temperature of the rheometer was set to 23.5 °C and equilibrated for at least 10 min. After adding the sample (*n* = 3) to the device and lowering the measuring head, the system was again equilibrated at 23.5 °C for 5 min. To assess the temperature‐dependency of the material behavior, the oscillation frequency was kept at a constant value of 1 Hz, and the temperature was first decreased from 23.5 to 10 °C at a constant rate of 0.5 °C min^−1^ and then increased to 30 °C at the same rate. The constant shear strain applied in those trials was set to the 1.5‐fold value of the averaged shear strain obtained in a pretest (applying the minimal torque of 0.5 µNm) at 23.5 °C. To determine the storage and loss moduli as a function of shear stress, shear stress sweeps from 0.01 to 10 000 Pa with oscillation frequency of 1 Hz were performed at temperatures between 22 and 25.5 °C after a 10 min equilibration. The yield stress was determined as the stress corresponding to the onset of the decrease in the storage modulus.

For the evaluation of the viscosity as a function of shear rate, a cone‐plate configuration (P‐PTD200 and CP25−1, Anton Paar) with a gap of 98 µm was used to perform shear rate sweeps from 1 to 1000 s^−1^ at each temperature between 22 and 25.5 °C after a 10 min equilibration.

### 4.2 Coconut Oil Extrudability Test

The coconut oil (bioasia, Sri Lanka) was brought to 37 °C and sterile filtered through a 0.2 µm syringe filter (Merck Millex, Darmstadt, Germany). Then, 3 cc syringes (EFD Nordson, Westlake, USA) were filled and stored at 4 °C for later use. The ink was printed with the temperature‐controlled print head mounted on the BioX printer (Cellink, Sweden) using a 27 G needle. The syringe was put in the incubator at 37 °C for 5 min to start warming it up. Then, it was inserted in the print head and left for 15 min at the set print head temperature prior to printing. The extrudability of the ink was tested at different print head temperatures (22–23.5 °C) and extrusion pressures of 330, 500, and 700 kPa by evaluating filament formation at the nozzle. The print head temperature was raised at 0.5 °C steps with a waiting time of 15 min at each step. Videos of the tests were recorded with an iPhone camera, and screen captures were taken from the videos.

### 4.3 Coconut Oil Printing

The effect of the *z*‐offset, print head temperature, nozzle speed, and extrusion pressure on the filament width was examined, by varying one parameter at the time. Straight lines were printed on glass slides and stored at 4 °C before imaging with the VHX‐5000 digital microscope (Keyence, Japan).

To investigate the effect of the *z*‐offset on the filament width, straight lines at *z*‐offsets between 0 and 200 μm were printed using a 27 G needle (*n* = 10 for each *z*‐offset) on glass slides. The *z*‐offset describes the vertical distance between the needle tip and the print bed after subtracting the needle inner diameter. The print head temperature was varied between 23.5 and 25.5 °C, the nozzle speed between 6 and 18 mm s^−1^, and extrusion pressure between 330 and 530 kPa. Straight lines were printed (*n* = 20 for each temperature, nozzle speed, and extrusion pressure), while keeping the other parameters constant.

To determine printing accuracy, a grid pattern with pore areas of 1, 4, 9, 16, and 25 mm^2^ consisting of two layers was printed at print head temperatures of 23.5, 24.0, 24.5, 25.0, and 25.5 °C (*n* = 3 for each temperature). The pore areas and perimeters were measured with the built‐in microscope software. The pore accuracy was calculated according to Equation (1) [65, 66], where *A*_t_ is the pore's theoretical area as defined in the G‐code with the target filament radius subtracted on each side, and *A*_a_ the pore's actual area.

(1) $${\text{Accuracy}}_{\text{pore}}=\left(\;\frac{A_{a}}{A_{t}}\;\right)\;\times 100\text{\%}$$

The optimized printing parameters for the 27 G needle included a print head temperature of 23.5 °C, nozzle speed of 18 mm s^−1^, a *z*‐offset of 150 μm, extrusion pressure of 330 kPa, and a bed temperature of 10 °C. These parameters were used for the following tests, unless stated otherwise.

The printing parameters for the 18 G needle included a print head temperature of 23.5 °C, nozzle speed of 10 mm s^−1^, a *z*‐offset of 650 μm, extrusion pressure of 55 kPa, and a print bed temperature of 10 °C. For the 20 G needle, the printing parameters included a print head temperature of 23.5 °C, nozzle speed of 5 mm s^−1^, a *z*‐offset of 400 μm, extrusion pressure of 110 kPa, and a bed temperature of 10 °C. For the 34 G needle, a print head temperature of 24.5 °C, nozzle speed of 2 mm s^−1^, a *z*‐offset of 50 μm, extrusion pressure of 700 kPa, and a print bed temperature of 10 °C were applied.

### 4.4 Fabrication of the Perfusion Chamber

A custom perfusion chamber was designed using Autodesk Inventor Professional 2023 (Autodesk Inc., San Francisco, USA). It consisted of a main part defining the hydrogel section embedding the microchannel, including the inlet and outlet, and two plates as top and bottom surfaces. The main part was additively manufactured by digital light processing (DLP) with an Asiga Max X43 printer (Asiga Europe, Erfurt, Germany) using the GR‐10 resin (pro3dure medical, Iserlohn, Germany), while the chamber top and bottom plates were cut out of a polymethyl methacrylate (PMMA) plate using a universal milling machine (Hermle U630T, Maschinenfabrik Berthold Hermle AG, Gosheim, Germany). Sealing was achieved with silicone membranes inserted in the predisposed grooves at the top and bottom surfaces of the main part and by screws fixing the plates to the main part. Connection of the chamber to the tubing was enabled by standard Luer connectors. The inlet and outlet of the chamber protruded into the hydrogel section with semicylindrical extensions onto which the ends of the coconut oil filament would be printed, in this way ensuring the connection to the microchannel. For cell experiments, all parts were washed with 70 % ethanol for an hour and subsequently were rinsed 3 times for 5 min with sterile PBS. For cell imaging or when using gelatin methacryloyl as the hydrogel matrix, the PMMA plates were exchanged for glass.

### 4.5 Hydrogel Molding

#### 4.5.1 Fibrin

Fibrin gel was prepared by mixing a fibrinogen and a thrombin solution. A frozen 20 mg ml^−1^ stock solution of fibrinogen from human plasma (Millipore Sigma, Merck KGaA, Darmstadt, Germany) was equilibrated to room temperature prior to use. A solution of 6 U ml^−1^ thrombin (Sigma–Aldrich, Germany) and 7.5 mM calcium chloride (Sigma–Aldrich, Germany) in tris‐buffered saline (TBS) (Sigma–Aldrich, Germany) was prepared separately. The TBS solution consisted of 27 mM tris hydrochloride (Sigma–Aldrich, Germany), 2.7 mM potassium chloride (Merck KGaA, Darmstadt, Germany), 137 mM sodium chloride (Sigma–Aldrich, Germany), and 5 mM tris base (Sigma–Aldrich, Germany) in ultrapure water (Berrytec, Germany), and the pH was set to 7.4. To obtain a fibrin gel, equal volumes of the thrombin and the fibrinogen solutions were mixed, dispensed in the mold, and incubated at room temperature for 45 min and then at 37 °C for 45 min.

#### 4.5.2 Gelatin

Gelatin gel was prepared by enzymatic crosslinking of gelatin. A solution of 10 wt% gelatin Type A (Sigma, Germany) in PBS (Gibco, Germany) was stirred at 90 °C for 4 h. After cooling down to 37 °C, the pH was adjusted to 7.4, and the solution was sterile filtered through a 0.2 µm syringe filter (Merck Millex, Darmstadt, Germany). A frozen transglutaminase (BDF Natural Ingredients, Girona, Spain) solution of 350 mg ml^−1^ in PBS was thawed and then activated at 37 °C for 20 min. Transglutaminase was added at a 1:20 volume ratio to the gelatin solution at 37 °C. The mixture was equilibrated to room temperature, dispensed in the mold, incubated at room temperature for 45 min, and then left to crosslink at 37 °C for 45 min.

#### 4.5.3 Gelatin Methacryloyl

Gelatin methacryloyl gel was prepared by UV crosslinking. A 5 wt% GelMA (Cellink, Sweden) was dissolved in PBS at 70 °C for 30 min. LAP photoinitiator (Cellink, Sweden) was dissolved in PBS separately at a concentration of 0.5 wt% at 70 °C for 30 min. Then, the GelMA and LAP solutions were mixed at 40 °C for 15 min. The pH was adjusted to 7.4 and the precursor solution was left to cool down to room temperature prior to molding. Crosslinking was performed for 60 min at 365 nm at room temperature.

#### 4.5.4 Fibrin/Gelatin

The following stock solutions were incubated at 37 °C for 30 min prior to use: a solution of 20 mg ml^−1^ human fibrinogen in TBS (Millipore Sigma, Merck KGaA, Darmstadt, Germany), a solution of 400 U ml^−1^ thrombin in TBS (Sigma–Aldrich, Germany), a solution of 15% gelatin type A in PBS prepared at 90 °C for 12 h (Sigma–Aldrich, Germany), a 50 mM calcium chloride solution in TBS (Sigma–Aldrich, Germany), and a 35% transglutaminase solution in PBS (BDF Natural Ingredients, Girona, Spain). A gel solution composed of 10 mg ml^−1^ human fibrinogen, 7.5 wt% gelatin, 2.5 mM calcium chloride, and 1% transglutaminase was prepared and kept at 37 °C for 90 min. Prior to molding, the gel solution was brought to room temperature, and thrombin (Sigma, Germany) was added to the solution at a final concentration of 10 U ml^−1^. The mixture was then dispensed in the mold, incubated at room temperature for 45 min, and then left to crosslink at 37 °C for 45 min.

### 4.6 Microchannel Fabrication

The perfusion chamber was assembled by screwing the PMMA bottom to the main part, and then the first gel layer with a volume of 700 µl was molded inside the chamber. Before printing, a UV cleaning cycle of the BioX printer was run, and the fan was started. The perfusion chamber was placed on the print bed, cooled down to 10 °C, and the channel pattern was printed directly on the first gel layer. The coconut ink was printed with a 27 G needle at a print head temperature of 23.5 °C, nozzle speed of 18 mm s^−1^, extrusion pressure of 330 kPa, 150 μm *z*‐offset. The second gel layer with a total volume of 700 µl was then molded on top of the first layer and the printed filament. The perfusion chamber was closed and time for crosslinking was allowed, as described in 4.5. Finally, a 5 ml syringe (Braun, Germany) was connected to the chamber luer inlet, and the ink was washed out with warm PBS at 37 °C.

### 4.7 Filament and Microchannel Width Evaluation

Microchannels (*n* = 4) were fabricated as described in 4.6 in fibrin gel. After washing with PBS, the channels were filled with blue dye. The printed filaments and subsequently the resulting perfused channels were imaged with a VHX‐5000 digital microscope (Keyence, Japan), and the widths were quantified using ImageJ 1.53e (Rasband, W.S., ImageJ, U.S. National Institutes of Health, Bethesda, Maryland, USA) using the plot profile analysis function of the gray value after setting the image type to 8‐bit. The swelling ratios were determined by dividing the channel width after filling with blue dye by the filament width after printing or by the filament width after molding the top fibrin layer.

### 4.8 Negative Replica of Microchannels

To visualize the channel cross‐section, a negative replica of the channel (*n* = 4) was created. A solution consisting of 40% (v/v) PEGDA (Sigma, Germany) and 0.5% (w/v) LAP (Cellink, Sweden) in PBS (Gibco) was prepared and stirred at 35 °C for 30 min at 300 rpm. The solution was stored at 4 °C in the dark until further usage. For visualization, the PEGDA was mixed with blue dye and used to fill a microchannel fabricated as described in 4.6. with a 2 ml syringe (Braun, Germany). The chamber was then closed with a glass cover, and UV crosslinking at 365 nm (1 mW cm^−2^) was performed for 3 h. After crosslinking, the chamber was opened, the fibrin gel was cut open with a scalpel, and the PEGDA negative replica of the channel was extracted and imaged with a VHX‐5000 digital microscope (Keyence, Japan). The width of the negative replica (*n* = 4 channels, *i* = 2) was measured with the built‐in microscope software.

### 4.9 Microchannel Cross‐Sectional Circularity

For the evaluation of the cross‐sectional circularity, microchannels (*n* = 4) were produced in a fibrin/gelatin matrix using a 20 G needle. The hydrogels were carefully cut with a scalpel at two different positions (4 and 8 mm from one end) along the channel's length to obtain tubular segments. These were positioned upright in well plates to visualize the cross‐section with a light microscope in phase contrast mode (BZ‐X800, Keyence, Japan). After setting the image type to 8‐bit in ImageJ 1.53e (Rasband, W.S., ImageJ, U. S. National Institutes of Health, Bethesda, Maryland, USA), the perimeter of the microchannel's cross‐section was manually traced by drawing a polygon. The “measure” function in ImageJ was used to determine the area and perimeter of the traced channel cross‐section. The circularity was then calculated by ImageJ according to Equation (2).

(2) $$\text{Circularity}=\frac{4\cdot \text{π}\cdot A}{P^{2}}$$

where *A* is the area and *P* the perimeter of the channel's cross‐section. A circularity value of 1.0 corresponds to a perfect circle. The diameter was determined using the plot profile function in ImageJ.

### 4.10 Cytotoxicity Assay

The cytocompatibility assay was conducted by creating extracts (*n* = 5) from the oil at different volume percentages in cell culture medium based on the extract test according to ISO 10993. After melting in the water bath at 37 °C for 30 min, the coconut oil was sterile filtered through a 0.2 µm syringe filter (Merck Millex, Darmstadt, Germany). To create the eluates, the oil was mixed with Dulbecco's Modified Eagle Medium (DMEM) (Gibco, USA) at concentrations of 5%, 10%, 30%, 50%, 70%, and 90% v/v. The oil/cell culture medium mixtures were incubated at 37 °C under stirring for 72 h. Afterwards, the mixtures were allowed to cool down at room temperature, and after the oil and medium separated, for each sample, the medium was retrieved from underneath the oil layer. The eluate of a latex glove was used as a positive cytotoxic control (PC), and untreated medium was used as a negative noncytotoxic control (NC). Hs27 fibroblasts (ATCC CRL‐1634, Germany) were seeded at a density of 10 000 cells cm^−2^ in a 96‐well plate (Greiner, Germany) and incubated in DMEM, supplemented with 10% fetal bovine serum (Gibco, USA) and 1% antibiotic‐antimycotic (Gibco, USA) at 37 °C in a humidified atmosphere containing 5% CO_2_. After 24 h, the medium was exchanged for the eluates for each sample group. Cell viability was determined at day 3 using a commercial XTT proliferation test kit (2,3‐bis‐(2‐methoxy‐4‐nitro‐5‐sulfophenyl)‐2H‐tetrazolium‐5‐carboxanilide, XTT, Roche, Basel, Switzerland) following the manufacturer's instructions. The absorbance was measured at 450 nm on a microplate reader (Spark, Tecan). The relative cell viability was determined by normalizing the measured absorbance values of the samples to that of the negative control.

### 4.11 Cell Proliferation Assay

The cell proliferation (*n* = 5) was analyzed by determining the metabolic activity of Hs27 fibroblasts using the cell proliferation kit II (XTT, Roche, Basel, Switzerland). Proliferation of Hs27 fibroblasts seeded at a density of 10 000 cells cm^−2^ was determined on controls and surfaces simulating the coconut ink channel printing and preparation process in a 96 well plate: (1) molded fibrin (Section 4.5) rinsed with PBS before cell seeding, (2) molded fibrin covered with 100 µl coconut ink at 37 °C and then rinsed with PBS before cell seeding, and (3) wells covered with 100 µl coconut ink at 37 °C and then rinsed with PBS before cell seeding. Cells seeded in wells with untreated medium were used as positive proliferation controls, and cells seeded in wells with DMEM containing 1% Triton X‐100 were used as negative nonproliferating controls. The XTT assay (Roche, Basel, Switzerland) was conducted according to the manufacturer's instructions on days 1, 2, 3, and 4. The absorbance was measured at 450 nm on a microplate reader (Spark, Tecan).

### 4.12 Endothelialization and Perfusion of the Microchannels

Human umbilical vein endothelial cells (HUVECs) were cultured in Endothelial Growth Medium 2 (EGM‐2, Lonza, Switzerland) supplemented with 2% fetal bovine serum, 0.2 µg ml^−1^ hydrocortisone, 4 ng ml^−1^ human fibroblast growth factor‐basic (hFGF‐b), 2 ng ml^−1^ vascular endothelial growth factor (VEGF), 5 ng ml^−1^ recombinant insulin‐like growth factor‐1 (R3‐IGF‐1), 75 µg ml^−1^ ascorbic acid, 10 ng ml^−1^ human epidermal growth factor (hEGF), 30 µg ml^−1^ gentamicin, 15 ng ml^−1^ amphotericin, 1 ng ml^−1^ heparin, and 1% antibiotic‐antimycotic (Gibco, USA). Microchannels were fabricated as described in 4.6 and washed with EGM‐2 medium. HUVECs were then seeded in the channels at a concentration of 350 000 cells cm^−2^. The EGM‐2 medium was supplemented with 178 µg ml^−1^ tranexamic acid (Carinopharm, GmbH, Eime, Germany) during the perfusion experiments. During the 6 h static incubation step, the chambers were rotated by 90° every 60 min. Afterwards, the channels were perfused at 0.1 dyne cm^−2^ on day 1 for 16 h overnight using the Reglo ICC peristaltic pump (Ismatec, Masterflex, Germany). On day 2, the shear rate was then increased in a stepwise manner over 2 h to 2 dyne cm^−2^ in steps of 0.5 dyne cm^−2^ every 30 min and maintained at 2 dyne cm^−2^ overnight for 16 h. On day 3, the shear rate was further increased stepwise over 8 h to 10 dyne cm^−2^ in steps of 1 dyne cm^−2^ every 60 min and maintained for 72 h. The endothelial cells were stained with calcein AM (Biomol, Germany) at a concentration of 2 μM in EGM‐2 medium for 15 min at 37 °C and images were taken with a fluorescence microscope BZ‐X800 (Keyence, Japan).

### 4.13 CD31 Staining

Channels (not previously stained with calcein‐AM) were fixed in a 4% formaldehyde solution in PBS (Carl Roth, Germany) for 15 min and then washed with PBS. For blocking, a PBS solution with 5% normal goat serum (Dako, Germany) was added into the channel and incubated for 60 min at room temperature. CD‐31 staining was performed with mouse anti‐human CD‐31 primary antibody (Sigma Aldrich, Germany) and goat‐anti‐mouse Alexa fluor 594 (Life Technologies, USA) secondary antibody, each for 1 h at room temperature. Nuclei were stained with a 0.2 µg ml^−1^ DAPI solution in PBS (Invitrogen, USA) for 5 min at room temperature. After each antibody incubation step and the DAPI incubation step, the channels were washed with PBS for 15 min. Images were taken with the fluorescence microscope BZ‐X800 (Keyence, Japan).

### 4.14 Actin Staining

Channels (not previously stained with calcein‐AM) were fixed in a 4% formaldehyde solution in PBS (Carl Roth, Germany) for 15 min and then washed with PBS for 15 min. For blocking and permeabilization, a PBS solution with 5% normal goat serum (Dako, Germany) and 0.1% Triton X‐100 (Sigma–Aldrich, Germany) was added into the channel and incubated for 60 min at room temperature. Actin staining was performed with Acti‐stain 488 Phalloidin (Cytoskeleton, Inc., Denver, CO, USA) at a concentration of 0.1 µM diluted in PBS for 1 h at 37 °C. Nuclei were stained with a 0.2 µg ml^−1^ DAPI solution in PBS (Invitrogen, USA) for 5 min at room temperature. After the actin and DAPI incubation step, the channels were washed with PBS for 15 min. The hydrogels were carefully cut with a scalpel at two different positions (4 and 8 mm from one end) along the channel's length (12 mm) to obtain tubular segments. These were positioned upright in well plates to visualize the cross‐section with the fluorescence microscope BZ‐X800 (Keyence, Japan).

### 4.15 Permeability Test of the Endothelialized Microchannels

Following the channel endothelialization and the perfusion step at 2 dyne cm^−2^ as described in 4.11, a FITC‐dextran solution (70 or 250 kDa, Sigma Aldrich, Germany) was prepared at a concentration of 0.5 mg ml^−1^ in EGM‐2 medium (Lonza, Switzerland) and was injected into endothelialized (*n* = 4) and nonendothelialized channels (*n* = 4) using a 2 ml syringe (Braun, Germany). The channels were imaged within the first 30 s after injection and after a 2 h static incubation at 37 °C. The fluorescence intensity (FI) was determined in the channel (region of interest: 200 × 800 µm) and in the matrix surrounding the channel on both sides (region of interest: 300 × 800 µm) using ImageJ 1.53e (Rasband, W.S., ImageJ, U.S. National Institutes of Health, Bethesda, Maryland, USA). The relative FI in the matrix was calculated as described in Equation (3).

(3) $$\text{Relative FI in the matrix}=\left(\;\frac{{\mathrm{FI}}_{\text{Hydrogel matrix}}}{{\mathrm{FI}}_{\text{Hydrogel matrix}}+\;{\mathrm{FI}}_{\text{channel}}}\;\right)\;\times \;100\text{\%}$$

### 4.16 Fabrication of 3D Constructs with Overhangs and Bridging Sections

The TUM logo (*n* = 3) was used as construct model featuring overhangs and bridging parts. The logo had a height of 20.6 mm, a width of 3.8 mm, and a length of 42.1 mm. Dual printing was achieved with two extrusion print heads, one for Pluronic F‐127 (Sigma–Aldrich, Germany) as structural ink and one for the coconut oil as sacrificial ink. The Pluronic F‐127 solution was prepared by dissolving 0.4 g ml^−1^ in cold water under stirring, which was filled in syringes and stored at 4 °C for later use. The printing parameters were as follows: extrusion pressure of 340 kPa, 35 °C print head temperature, and a speed of 6 mm s^−1^. The coconut oil was printed at 23.5 °C print head temperature, nozzle speed of 18 mm s^−1^, and extrusion pressure of 330 kPa. For both materials, a 27 G needle (Cellink, Gothenburg, Sweden) was used. The print bed temperature was set to 17.5 °C. After printing, the construct was removed from the print bed, and the coconut oil was melted on a heating plate at 27 °C for 15 min. The height and length of the entire construct (*n* = 3) and the widths of the legs at 3 different positions (*i* = 3) were measured directly after melting the oil support structures. The accuracy of the logo was determined by dividing the actual dimensions (*x*_a_) by the theoretical dimensions (*x*_t_) as described in Equation (4).

(4) $$\text{Accuracy}=\left(\;\frac{x_{a}}{x_{t}}\;\right)\;\times \;100\text{\%}$$

### 4.17 Embedded Printing in Coconut Oil‐Based Support Bath

#### 4.17.1 Support Bath Preparation

A total of 180 ml coconut oil was mixed with 54 ml of a 1% (w/v) xanthan gum solution dissolved in deionized water. The mixture was stirred for 3 min using a handheld milk frother (Philorn Technology Co.) at 23 000 rpm. The bath was centrifuged at 1750 rpm for 3 min, and the supernatant was discarded. Calcium chloride (Sigma–Aldrich, Germany) was added at a final concentration of 4 mM, and the bath material was vortexed for 1 min. Depending on the sizes of the printed constructs, Petri dishes or wells in a 6 or 12 well plate were filled with the support bath, and placed on the print bed of the BioX printer at room temperature.

To visualize the microspheres of the support bath, a droplet of the bath material (*n* = 5) was diluted in 5 ml distilled water, and 20 µl was pipetted and spread on a glass slide. After covering the diluted bath suspension on the glass slide with a cover glass, the microspheres were imaged with the BZ‐X800 Keyence microscope.

#### 4.17.2 Printing Parametric Study for Alginate Filaments

A 4% (w/v) solution of sodium alginate (Kimica Lagin, Grade I‐1G) was prepared by mixing alginate in distilled water overnight at room temperature using a magnetic stirrer at 400 rpm until the alginate powder completely dissolved. Before printing, the alginate was transferred into a 3 ml syringe (Nordson, Westlake, USA) and centrifuged at 100 g for 5 min to remove air bubbles. Different nozzles—20, 22, 25, 27, 30, and 32 G—were used to print alginate filaments of 2 cm length (*n* = 5) by applying a nozzle speed of 14 mm s^−1^ and an extrusion pressure of 440, 250, 150, 120, 50 and 35 kPa, respectively. The filament widths were quantified using the digital microscope's software (VHX‐5000, Keyence, Japan).

#### 4.17.3 Printing of Alginate‐Based Hollow Cylinders

Hollow cylinders (*n* = 5) of 10 mm diameter and 3 mm height were printed at a *z*‐offset of 2 mm from the well plate bottom, with a 32 G needle, at a nozzle speed of 14 mm s^−1^, and an extrusion pressure of 440 kPa. The constructs were left to crosslink for 30 min at room temperature, and the well plate was placed in the incubator for 30 min to melt the support bath. All constructs were removed from the bath, incubated in PBS (Gibco, USA) at 37 °C to melt any residual bath material for 30 min. The constructs were stored in PBS at 4 °C until they were imaged with the VHX‐5000 digital microscope (Keyence, Japan).

#### 4.17.4 Printing of a Gelatin‐Based Carotid Sinus

An 8 wt% gelatin solution was prepared by dissolving gelatin type A (Sigma, Germany) in PBS and stirring at 70 °C for 16 h. pH was then adjusted to 7.4 at 37 °C. To better visualize the printed construct inside the support bath, purple food color (Dr. Oetker, Germany) was added to the gelatin solution in a 1:100 dilution, before filling the printing syringe. For printing the carotid sinus model from gelatin, the prepared bath was supplemented with 0.35% transglutaminase (BDF Natural Ingredients, Girona, Spain). The G‐code for a carotid sinus model (free CAD model available on grabcad.com/library/carotid‐sinus‐1) was generated using Repetier Host (Hot‐World GmbH & Co. KG). The model was scaled down to a height of 24 mm and a wall thickness of 250 µm and printed in a 100 ml urine beaker (Sarstedt AG & Co.KG, Nürnbrecht, Deutschland) using a 27 G nozzle. An offset from the beaker bottom of 2 mm, an extrusion pressure of 130 kPa, a print head temperature of 26 °C, a nozzle speed of 3 mm s^−1^, and a preflow time of 200 ms were applied. The construct was crosslinked for 30 min in the bath at room temperature, and then the beaker was placed in the incubator for 30 min to melt the support bath. After removal from the bath, the construct was incubated in PBS (Gibco, USA) at 37 °C to melt any residual bath material for 30 min and then imaged with the VHX‐5000 digital microscope (Keyence, Japan).

### 4.18 Statistical Analysis

The results are expressed as the mean ± standard deviation. The Shapiro–Wilk test and Lilliefors test were used to test for normal distribution, and a two‐sample F‐test was employed to check for equal variances. The unpaired two‐sided t‐test between the groups with normal distribution was conducted, when homogeneity of variances was met, and for samples with unequal variances, the unpaired two‐sided t‐test with Welch's correction was used. The Mann–Whitney test was applied for samples without normal distribution. Statistically significant level was determined at *p* < 0.05. All the data were analyzed using GraphPad Prism 9.

## Funding

This study was supported by TUM's Innovation Network ARTEMIS (Number: N2001) and Förderlinie Development Grant from Forschungsallianz Bayern—Queensland (Number: DEV2024_1_14).

## Ethics Statement

Ethics committee approval and informed consent from volunteers have been obtained. Human‐derived perinatal cells from healthy volunteers are used according to the ethics vote of the Technical University of Munich ethics committee: 2023‐530‐S‐KH.

## Conflicts of Interest

The authors declare no conflicts of interest.

## Supporting information

Supplementary Material

## Data Availability

The data that support the findings of this study are available from the corresponding author upon reasonable request.
