# Supplementary material for: Coconut Oil as Lipid‐Based 3D‐Printable Sacrificial Material for the Facile Biofabrication of Precise Perfusable Microchannels and Freeform Hydrogel Structures
Source: Small Sci. 2026 Aug 19;6(8):e70371. doi: 10.1002/smsc.70371 (PMC13489305; doi:10.1002/smsc.70371)
Supplement: Supplementary file 1 [file SMSC-6-e70371-s001.pdf]

Supporting Information

Coconut oil as lipid-based 3D printable sacrificial material for the facile biofabrication of precise perfusable microchannels and freeform hydrogel structures

*Salma Mansi, Susanne Halbherr, Franziska Möller, Anna Lotta Ernst, Sophie Bauer, Zinaida Kuznetsova, Myrto Karagiannakidou Samsarelou, Carolin A. Rickert, Dominik Heinrich, Zhaoyang Zhong, Diana M. Rojas-González, Oliver Lieleg, Petra Mela**

Content

Methods
1. Cell proliferation
2. Endothelial cell density and orientation under dynamic culture in microchannels

Results
3. Rheological characterization
4. Extrudability test
5. Cell proliferation fluorescence images
6. Endothelial cell orientation and density in microchannels under dynamic cultivation
7. Permeability test of the endothelialized microchannels
8. Evaluation of microchannel endothelialization
9. Printing of large and high aspect ratio constructs
10. Effect of print bed temperature on the filament width
11. Comparison of different coconut oil products

Methods

1. Cell proliferation

Proliferation of Hs27 fibroblasts seeded at a density of 10,000 cells cm^{-2} was determined on controls and surfaces simulating the coconut ink channel printing and preparation process in a 96 well plate: 1. molded fibrin washed with PBS before cell seeding, 2. molded fibrin covered with 100 µl coconut ink and washed with PBS before cell seeding, and 3. wells covered with 100 µl coconut ink and washed with PBS before cell seeding. Cells seeded in wells with untreated medium were used as positive proliferation controls. On days 1 – 4, the cells were fixed and stained for fluorescence imaging. Briefly, the samples were fixed in a 4 % formaldehyde in PBS solution (Carl Roth, Germany) for 30 minutes and then washed with PBS. Blocking and permeabilization was performed with PBS containing 5 % normal goat serum (Dako, Germany) and 0.1 % Triton X-100 (Sigma, Germany) for 60 minutes at room temperature. Samples were stained for actin using Acti-stain phalloidin 488 (Biozol, Germany) for 1 hour at 37 °C, and nuclei staining was conducted with DAPI (Invitrogen, USA) for 5 minutes at room temperature and then washing with PBS for 15 minutes. Fluorescence images were taken with the fluorescence microscope BZ-X800 (Keyence, Japan).

2. Endothelial cell density and orientation under dynamic culture in microchannels

Cell density was quantified by counting DAPI-stained nuclei using ImageJ 1.53e (Rasband, W.S., U.S. National Institutes of Health, Bethesda, MD, USA). Fluorescence images were first converted to 8-bit grayscale. The Analyze Particles function was then used to automatically count the nuclei. Then, the cell number was divided by the surface area to provide the cell density. Cell orientation was evaluated using the OrientationJ plugin in ImageJ by determining the distribution of the orientation angles of the DAPI-stained nuclei. Prior to the analysis, the images were converted to 8-bit grayscale. OrientationJ was used to calculate the local orientation of each pixel for all nuclei, and the orientation angle distribution was then normalized to the total pixel count for all orientation angles. The orientation angle was defined relative to the longitudinal axis of the channel, with 90° corresponding to the channel axis and flow direction, and 0° and 180° corresponding to the direction perpendicular to the flow.

Results

3. Rheological characterization

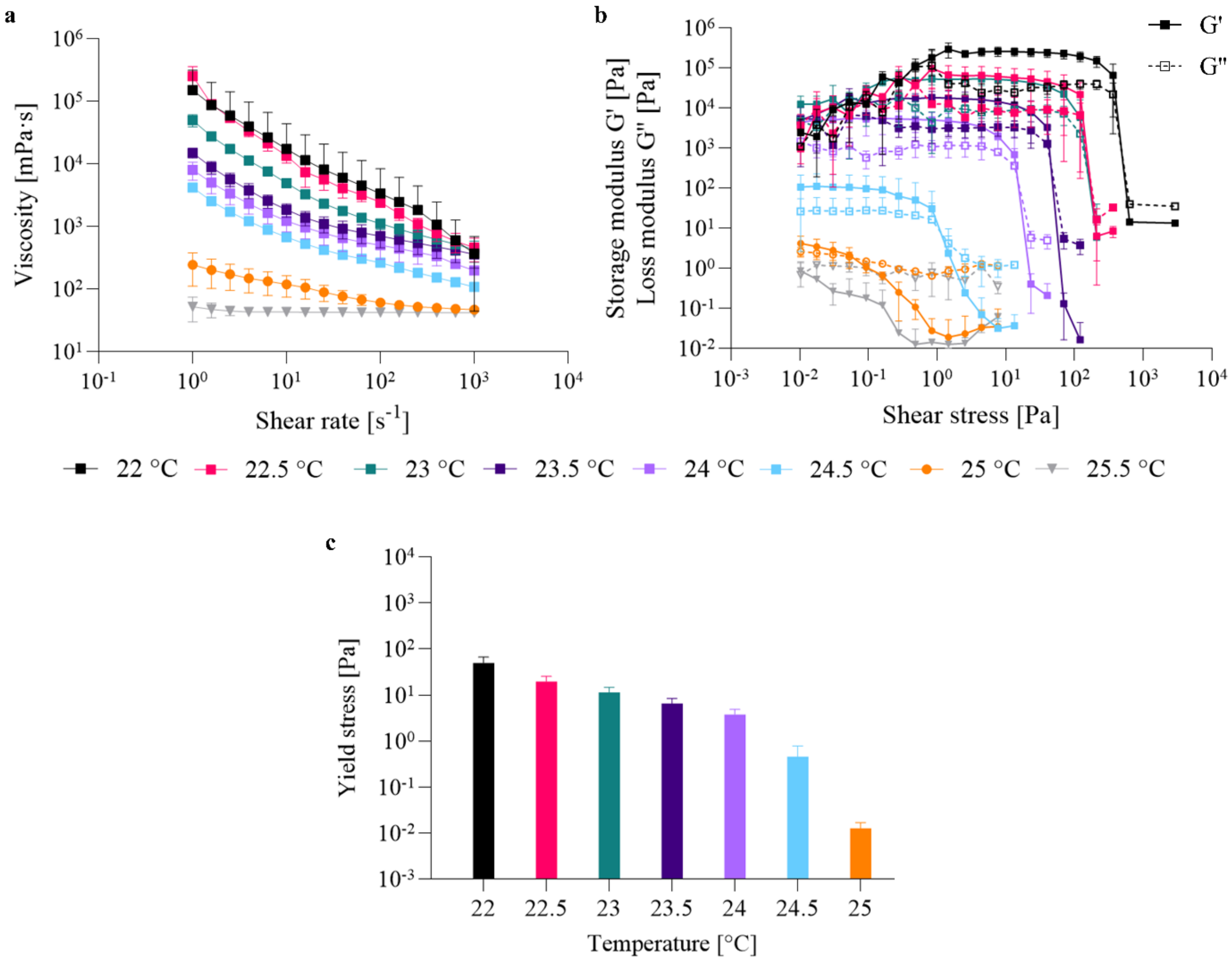

Figure S1. Rheological characterization at temperatures between 22 and 25.5 °C. **a)** Viscosity as a function of shear rate. **b)** Storage modulus (G′) and loss modulus (G″) as a function of shear stress at a frequency of 1 Hz. **c)** Yield stress evaluation from the shear stress ramp. **d)** Viscosity as a function of time at different shear rates measured at the printing temperature (23.5 °C). Values represent mean ± standard deviation as obtained from n = 3 independent samples.

4. Extrudability test

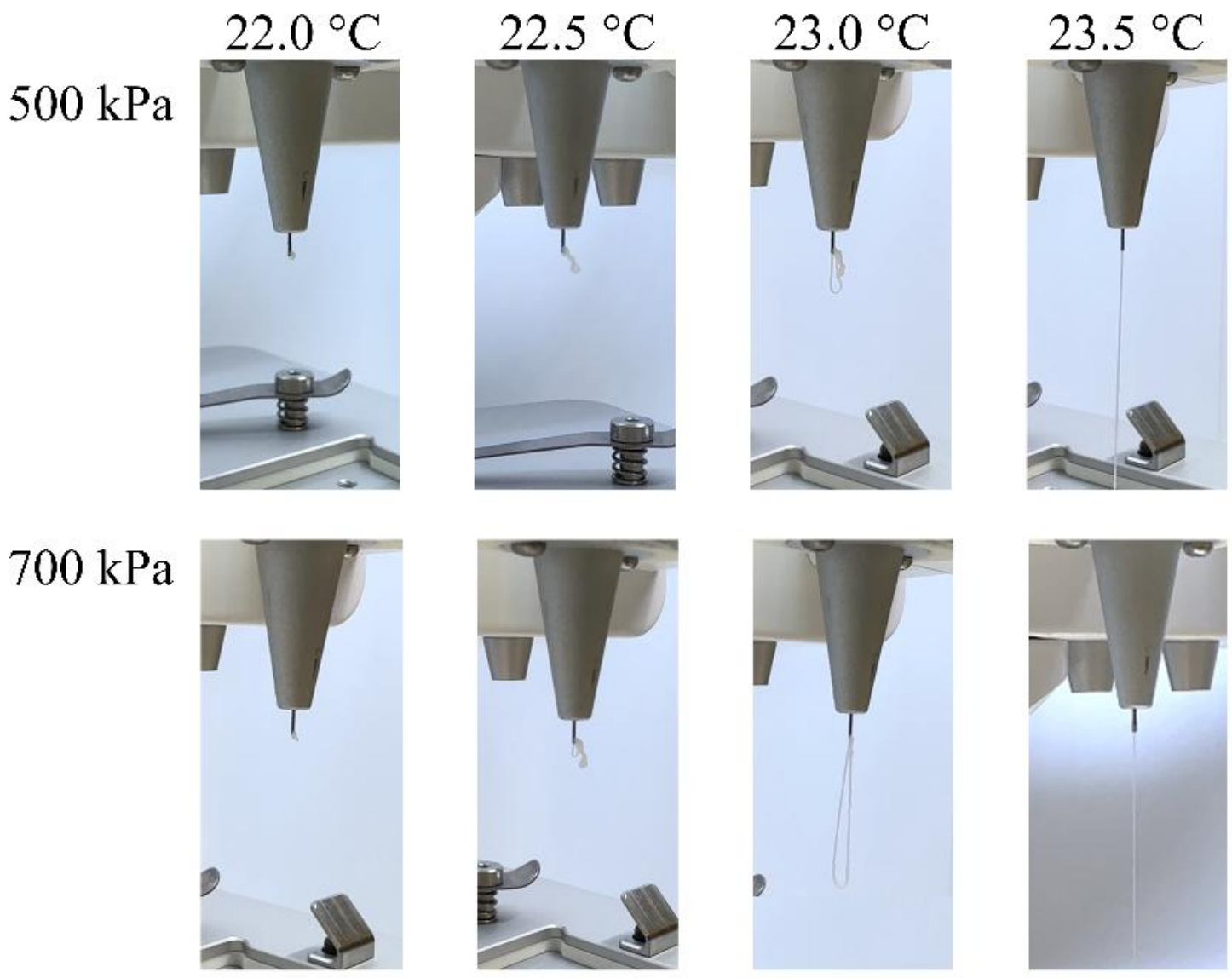

Figure S2. Ink extrudability test. The extrudability of the coconut oil was tested at different print head temperatures (22 – 23.5 °C) and extrusion pressures (500 and 700 kPa).

5. Cell proliferation fluorescence images

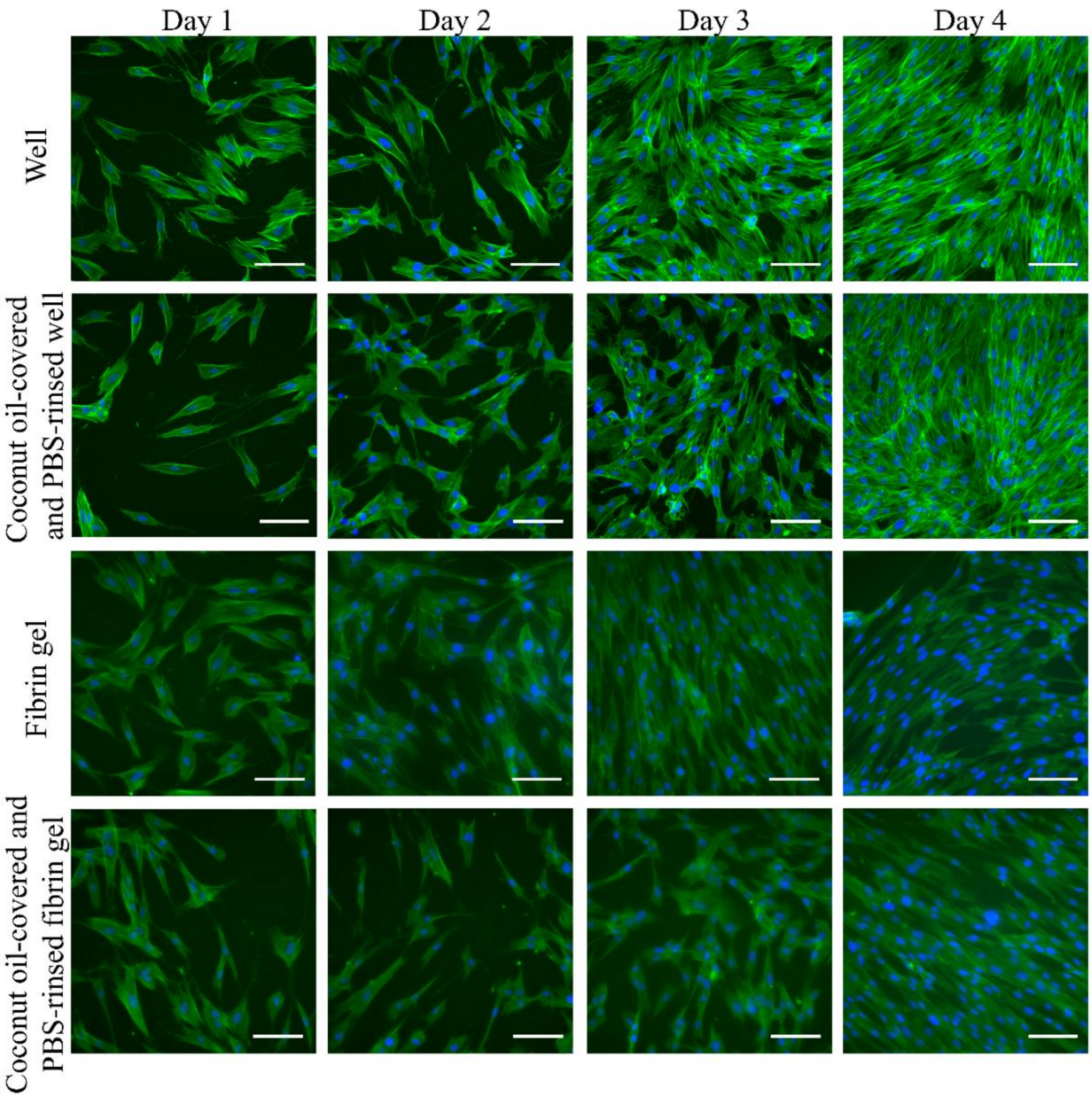

Figure S3. Proliferation study. Fluorescence images of Hs27 fibroblasts seeded on previously oil-covered and PBS-rinsed surfaces as well as untreated surfaces over 4 days, stained for actin (green) and DAPI (blue). Scale bar: 100 µm.

6. Endothelial cell orientation and density in microchannels under dynamic cultivation

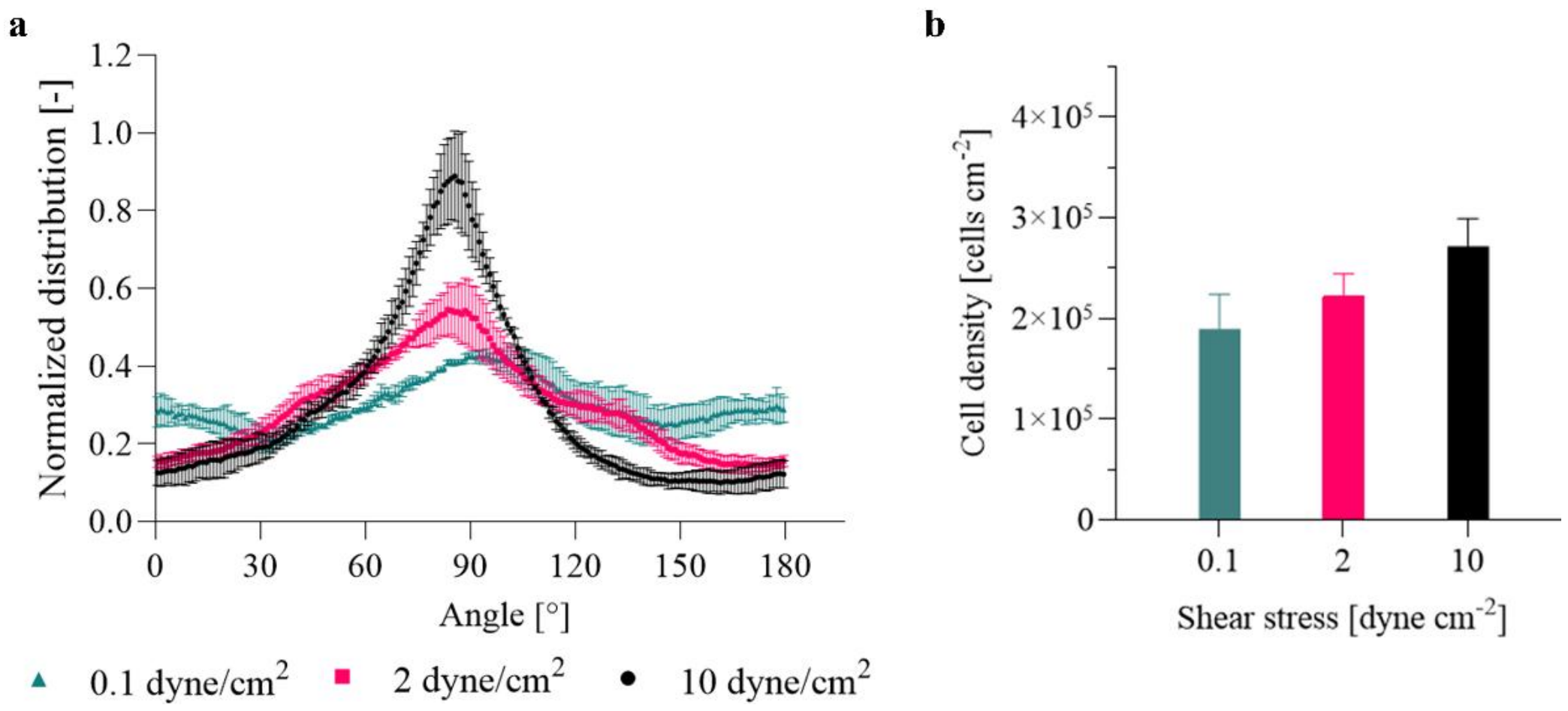

Figure S4. Dynamic cultivation of endothelialized microchannels. **a)** Normalized distribution of endothelial cell nuclear orientation and **b)** quantification of the endothelial cell density under shear stresses of 0.1, 2, and 10 dyne cm^{-2}. 90° represents the longitudinal axis of the channel, parallel to the flow direction. Values represent mean ± standard deviation as obtained from n = 3 independent samples.

7. Permeability test of the endothelialized microchannels

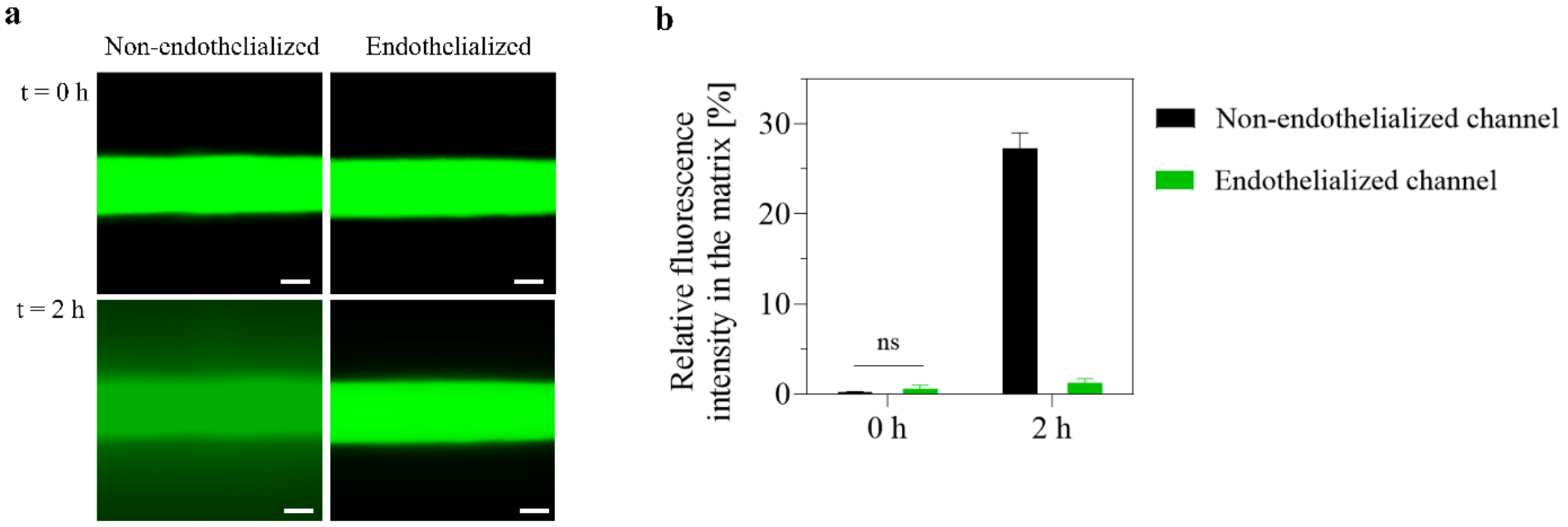

Figure S5. Permeability test of the endothelialized microchannels. **a)** Images of the non-endothelialized and endothelialized channels incubated with 250 kDa FITC dextran at 0 and 2 hours. The fluorescence intensity was determined in the matrix surrounding the channel and inside the channel to determine **b)** the relative fluorescence intensity of the endothelialized and non-endothelialized channels. Values represent mean ± standard deviation obtained from n = 4 independent experiments. Scale bar **a**: 100 µm.

8. Evaluation of microchannel endothelialization

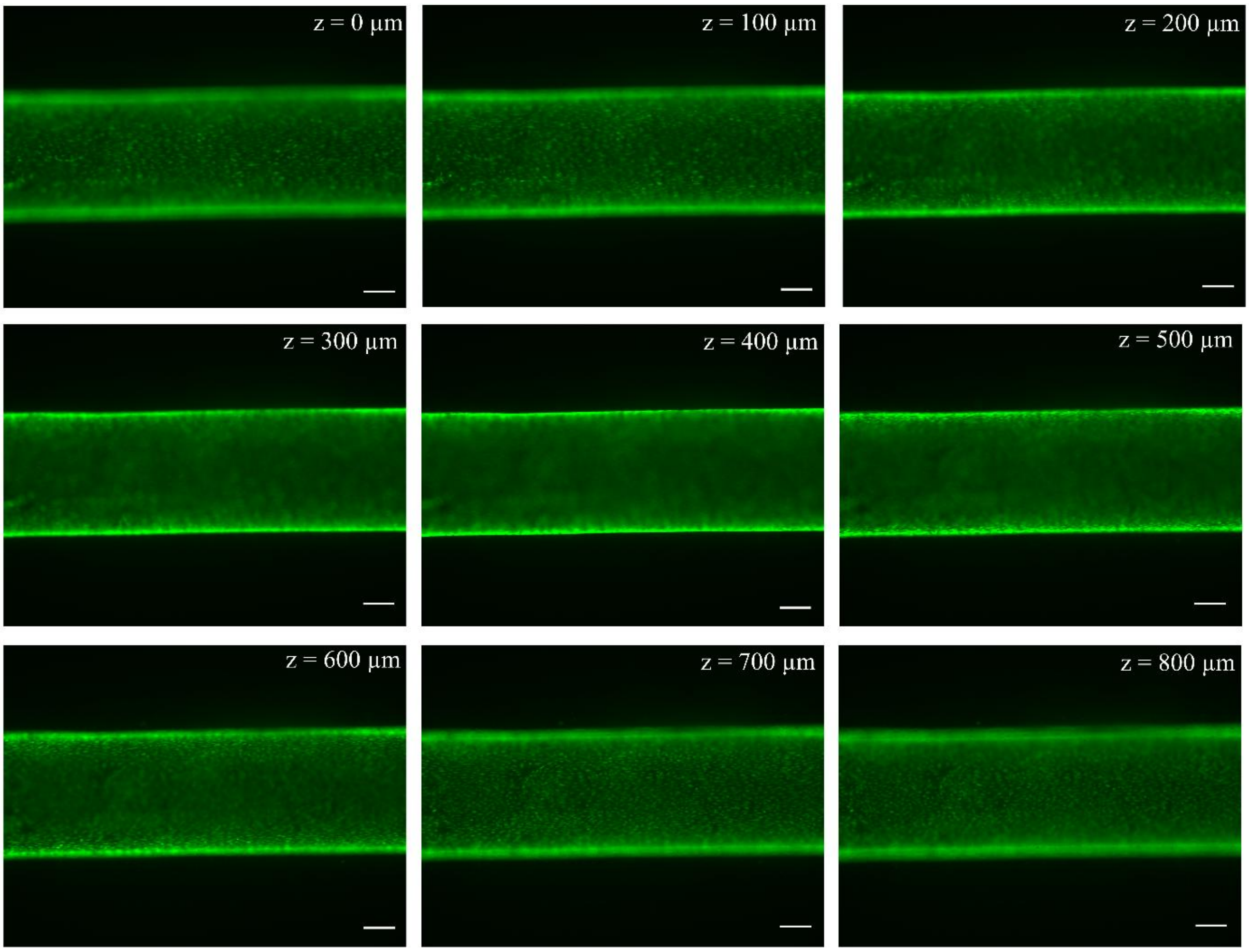

Figure S6. Evaluation of microchannel endothelialization. Fluorescence images of an endothelialized microchannel stained with calcein-AM taken at different z-positions. The microchannel was produced in a fibrin/gelatin matrix with a18 G needle. Scale bar: 200 µm.

9. Printing of large and high aspect ratio constructs

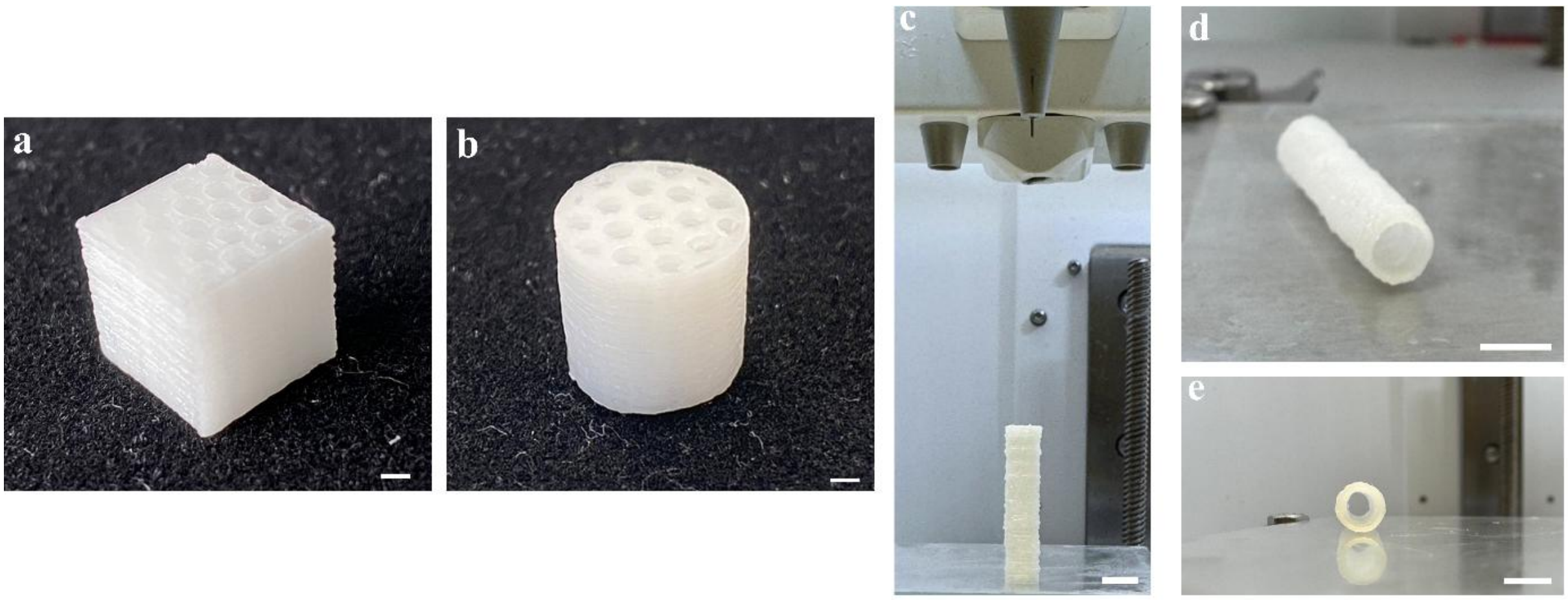

Figure S7. Printing of large and high aspect ratio constructs. Images of a printed **a)** 10 x 10 x 10 mm cube and **b)** a 10 mm diameter × 10 mm height cylinder, both with a 15 % honeycomb infill (27 G needle, 23.5 °C print head temperature, 18 mm s^{-1} nozzle speed, 150 μm z-offset, 330 kPa extrusion pressure, and of 10 °C bed temperature); **c-e)** Images of a printed hollow cylinder with a height of 22 mm, an outer diameter of 5 mm and wall thickness of 0.6 mm (20 G needle, 23.5 °C print head temperature, 5 mm s^{-1} nozzle speed, 400 μm z-offset, 110 kPa extrusion pressure, and of 10 °C bed temperature).Scale bar: **a-b**: 2 mm, **c-d**: 5 mm.

10. Effect of print bed temperature on the filament width

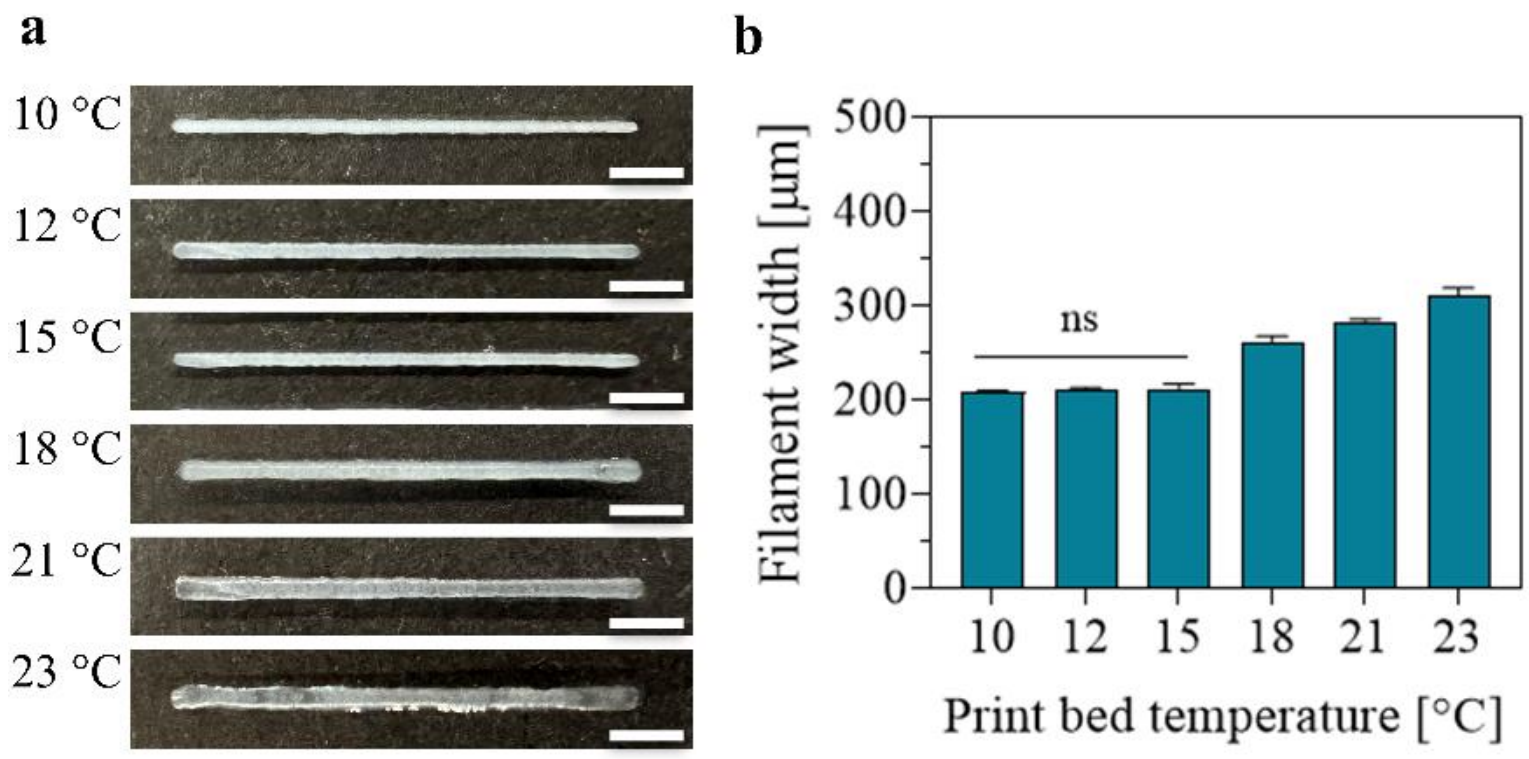

Figure S8. Effect of the print bed temperature. **a)** Visualization and **b)** quantification of the filament width obtained with a 27 G needle as a function of the print bed temperature. A print head temperature of 23.5 °C, nozzle speed of 18 mm s^{-1}, a z-offset of 150 µm, and an extrusion pressure of 330 kPa were applied. Values represent mean ± standard deviation as obtained from n = 5 independent samples. Scale bar: 1 mm.

11. Comparison of different coconut oil products

Table S1. Printability comparison of different coconut oil products.

	Product	Nozzle gauge	Print head temperature [°C]	Print bed temperature [°C]	Extrusion pressure [kPa]	Nozzle speed [mm s^{-1}]	Diameter [µm]
a	Alnatura organic coconut oil neutral	27 G	23.3	10 °C	500	12	220.2 ± 16
b	CocoNativo BioKokosöl deodorized organic coconut oil	27 G	22.8	10 °C	600	4	210.3 ± 11
c	GreatVita virgin cold-pressed organic cocounut oil	27 G	23.5	10 °C	700	2	215.3 ± 18
d	Monte Nativo coconut cold-pressed oil organic	27 G	23.1	10 °C	310	13	216.9 ± 24
e	KTC 100% pure coconut oil premium quality	27 G	23.1	10 °C	400	16	223.1 ± 6
f	Natur Planet virgin unrefined coconut oil	27 G	23.1	10 °C	540	8	211.2 ± 7

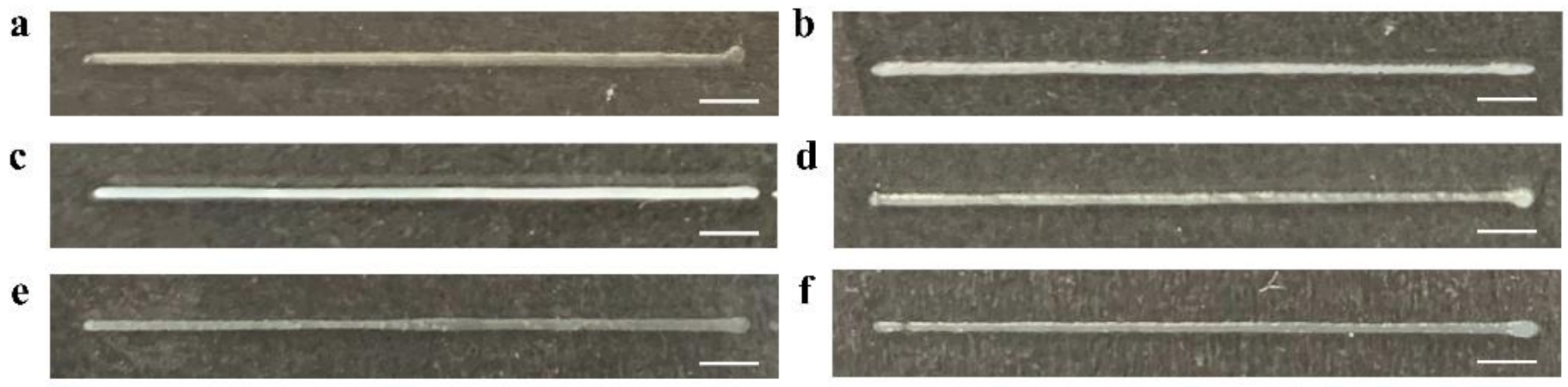

Figure S9. Comparison of different coconut oil products. Images of filaments printed from **a)** Alnatura organic coconut oil neutral, **b)** CocoNativo Bio Kokosöl deodorized organic coconut oil, **c)** GreatVita virgin cold-pressed organic coconut oil, **d)** Monte Nativo cold-pressed organic coconut oil, **e)** KTC pure coconut oil, and **f)** Natur Planet virgin unrefined coconut oil, using a 27 G needle and applying a print bed temperature of 10 °C. Scale bar: 1 mm..
